# Structure Engineering of Ni/SiO_2_ Vegetable Oil Hydrogenation Catalyst via CeO_2_

**DOI:** 10.3390/ijms25147585

**Published:** 2024-07-10

**Authors:** Margarita Gabrovska, Dimitrinka Nikolova, Vojkan Radonjić, Daniela Karashanova, Aleksandra Baeva, Tsvetomila Parvanova-Mancheva, Peter Tzvetkov, Evangeliya Petrova, Gabriella Zarkova, Jugoslav Krstić

**Affiliations:** 1 Institute of Catalysis, Bulgarian Academy of Sciences, 1113 Sofia, Bulgaria; margo@ic.bas.bg (M.G.); or mila_parvanova@abv.bg (T.P.-M.); evpetrova@ic.bas.bg (E.P.); gzarkova@ic.bas.bg (G.Z.); 2 Institute of Chemistry, Technology and Metallurgy, Department of Catalysis and Chemical Engineering, University of Belgrade, 11006 Belgrade, Serbia; vojkan.radonjic@ihtm.bg.ac.rs; 3 Institute of Optical Materials and Technologies, Bulgarian Academy of Sciences, 1113 Sofia, Bulgaria; dkarashanova@yahoo.com; 4 Institute of General and Inorganic Chemistry, Bulgarian Academy of Sciences, 1113 Sofia, Bulgaria; baeva@svr.igic.bas.bg (A.B.); tzvetkov@svr.igic.bas.bg (P.T.)

**Keywords:** Ni/SiO_2_ catalysts, CeO_2_ doping effect, SEM-EDS, HRTEM, partial hydrogenation of sunflower oil, fatty acids composition

## Abstract

Inspired by our finding that metallic Ni particles could be uniformly distributed on a reduced CeO_2_ surface and stabilized on Ce^3+^ sites, we suppose a possible improvement in the activity and selectivity of the MgNi/SiO_2_ vegetable oil hydrogenation catalyst by increasing the surface metal Ni availability via modification by ceria. The proposed approach involved the addition of a CeO_2_ modifier to the SiO_2_ carrier and as a catalyst component. Evaluation of the structure, reducibility, and surface and electronic states of the CeO_2_-doped MgNi/SiO_2_ catalyst was performed by means of the Powder X-ray diffraction (PXRD), Scanning electron microscopy-energy dispersive spectroscopy (SEM-EDS), and X-ray photoelectron spectroscopy (XPS) combined with High-resolution transmission electron microscopy (HRTEM), Temperature-programmed reduction with hydrogen (H2-TPR), and H_2_-chemisortion techniques. So far, no studies related to this approach of designing Ni/SiO_2_ catalysts for the partial hydrogenation of vegetable oil have been reported. The added ceria impact was elucidated by comparing fatty acid compositions obtained by the catalysts at an iodine value of 80. In summary, tuning the hydrogenation performance of Ni-based catalysts can be achieved by structural reconstruction using 1 wt.% CeO_2_. The introduction mode changed the selectivity towards C18:1-*cis* and C18:0 fatty acids by applying ceria as a carrier modifier, while hydrogenation activity was improved upon ceria operation as the catalyst dopant.

## 1. Introduction

Although discovered by the end of the 19th century, the hydrogenation of vegetable oils continues to be the most relevant catalytic process for the modification of fats and oils in the modern food industry [1,2,3,4,5,6]. The process involves the conversion of liquid oil into a solid or semisolid product by a catalytic reaction with a hydrogen.

Potential applications of catalysts developed for the hydrogenation of vegetable oils extend beyond the food industry. Various non-food sectors such as cosmetics [7], pharmaceuticals [8], paints and coatings [9], plastics [10], inks [11], adhesives [12], and textiles [13] also utilize hydrogenated oils for their unique properties. These industry sectors could benefit significantly from the novel materials and catalysts described in the article. For instance, in cosmetics and personal care products, hydrogenated oils are valued for their moisturizing and emollient properties. In pharmaceuticals, they serve as excipients and carriers in drug formulations. Additionally, in paints, plastics, and adhesives, hydrogenated oils enhance product durability, flexibility, and performance. Another significant application of hydrogenated vegetable oil is in the production of biofuels. As society strives towards sustainable energy sources, hydrogenated oils play a crucial role due to their ability to serve as a feedstock for biodiesel production [14,15].

Vegetable oil hydrogenation processes and the resultant products are becoming increasingly important given their low ecotoxicity and high biodegradability.

Common vegetable oils consist of fatty acid (FA) triglycerides of different compositions and degrees of unsaturation. The majority of unsaturated FAs contain C18:3 linolenic acid, C18:2 linoleic acid, and C18:1 oleic acid that are originally in the *cis*-configuration. Some carbon double bonds in *cis* form are isomerized into *trans* during hydrogenation. As a result, hydrogenated oil contains *cis*-FAs, *trans* fatty acids (TFAs), and a certain amount of saturated fatty acids (SFA), mainly C16:0 palmitic acid and C18:0 stearic acid. Highly unsaturated oils such as soybean and sunflower are very sensitive to oxidation, which causes rancidity during storage and, accordingly, reflects on their taste. Considering the relative oxidation rate of (C18:1):(C18:2):(C18:3) is 1:40:100, it is critical to manufacture stable hydrogenated oil containing the predominantly maximum amount of C18:1 and a significantly reduced content of C18:2, TFAs, and C18:0 acid. Oxidative stability is an important indicator to determine oil quality and shelf life. The particular interest in oleic acid is based on its stability in an oxygen environment, thus preventing spontaneous polymerization, as it remains liquid even at low temperatures [16,17].

Hence, the first goal of the hydrogenation process is to enhance the stability of the resulting solid or semisolid products at room temperature by decreasing FA unsaturation. The second goal is to improve the utility by altering the melting and solidification features of the oils [5]. Managing the FA composition, increasing the chemical and thermal stability, and achieving the desired properties of the vegetable oil’s derivatives are attained by partial hydrogenation of the unsaturated bonds, which enables usage of the obtained products for human consumption. They are employed as precursors with culinary properties for the production of margarine, cooking, frying and salad oils, mayonnaise, chocolates, ice creams, and different products of baking. The catalytic partial hydrogenation of vegetable oils is therefore currently the focus of scientific attention [17,18,19,20].

The renewed worldwide scientific interest in the process is related to the increasingly strict requirements regarding FA composition in edible hydrogenated oils. Animal products and some plant foodstuffs represent the main sources of SFAs in human food. A relative risk of SFA intake has been stated for the occurrence of coronary heart disease, stroke, some types of cancer, and cardiovascular disease [21]. The impact of TFAs on health is more complicated, since different types of TFAs should be considered. As has been reported by Aldai et al. [22], an important source of TFAs in the human diet are industrially produced iTFAs, which are formed during the catalytic partial hydrogenation of vegetable oils and thus found in different food products. Another source of TFAs are naturally occurring nTFAs, which are produced in the rumen of ruminants by the bacterial transformation of unsaturated FAs gained from the feed. Consequently, animal products contain nTFAs. The industrially produced oils may contain up to 60% TFAs compared to the content in ruminant fat, which generally does not exceed 6% [23]. As claimed by Pipoyan et al. [24], iTFAs are more deleterious than ruminant-origin TFAs. However, the latter are also considered to have some adverse health effects in certain cases. Various epidemiologic and clinical studies have provided strong evidence and confirmed the adverse role of TFAs in human health associated with an increased risk of oncologic, cardiovascular, and coronary diseases, as well as diabetes [24,25]. However, TFAs cannot be eliminated from the human diet, since they are present in meat and dairy products of ruminants [26].

Regardless of the source, the existence of SFAs and TFAs in human food is undesirable in accordance with the requirements of nutrition science. This is the main reason for the increased social attention to the consumption of hydrogenated fats and oils. In this context, the World Health Organization recommends limiting SFAs in food to a maximum of 10 wt.% and TFAs to a maximum of 1 wt.% or as low as possible, aiming to reduce the risk primarily of ischemic heart disease and stroke [27]. This stimulated the European Union to request less than 2 wt.% of TFAs in food products [28].

To achieve the foregoing targets, researchers should consider supported metal catalyst types, carrier structure, and metal–support interaction, as well as operating parameters such as reaction temperature, hydrogen pressure, and stirring rate [29].

Industrial partial hydrogenation of vegetable oils with hydrogen gas in the presence of the metal nickel supported on different natural sources of silica is well established worldwide. Commonly, the process is realized in conventional slurry reactors operating in the batch or semi-batch mode in the temperature range of 150–200 °C, hydrogen pressure between 0.1 and 0.6 MPa, and catalyst loading from 0.01 to 0.2 wt.%, depending on the properties of the final product [3,5].

The catalyst type is still a subject of interest and investigation due to the catalyst’s role in binding the H_2_ molecule and facilitating the reaction between hydrogen and the FA molecules. Researchers’ efforts have been focused on developing noble metals as an alternative to Ni catalysts to reduce the amount of TFAs [30,31]. Noble metals were found to exhibit the highest activity and selectivity to the formation of *cis*-monounsaturated FAs in this order: Rh > Pd > Pt > Ru > Ni. A similar trend was observed in terms of selectivity towards TFA production: Rh > Pd > Ru > Ni > Pt [32]. Regardless of the exceptional activity and TFA selectivity, the industrial application of noble metal catalysts is limited due to excessive prices and restricted resources. Cu-containing catalysts produced low levels of TFAs and were examined as less expensive alternatives to noble catalysts, but metallic Cu tends to dissolve in the oil, stimulating FA oxidation [3].

From a practical standpoint, supported Ni catalysts will be preferable at the industrial scale due to the high hydrogenation activity, inert nature relative to vegetable oil, easy separation from processed oil, and economic price, which manifest superiority over other metals [5]. Being a transition metal, nickel’s low cost and availability outlined the focus of our topical studies to develop active and selective Ni-based catalysts for the partial hydrogenation of vegetable oil, applying a diatomite carrier as a natural SiO_2_ source [33] and alternative silica gel as a commercial silica product [34,35,36]. In these studies, an attempt was made to elucidate the carrier impact on Ni catalyst reducibility and activity for vegetable oil hydrogenation, the latter being synthesized using different silica sources having different texture parameters. It was established that the origin of the SiO_2_ support and Mg dopant have an influence on the surface properties, reducibility, activity, and selectivity of the catalysts.

Despite many efforts of researchers to minimize TFA formation in the hydrogenated products, it remains the main challenge in the food industry [20,37]. To accomplish this task, the addition of a proper promoter or modifier to the Ni-containing catalysts would be a beneficial approach [38,39]. Ceria (CeO_2_) is one of the most extensively studied and employed reducible catalyst supports and promoters [40]. As a support for Ni catalysts, ceria is of special interest due to the ability of the Ce cation to change its formal oxidation state from Ce^4+^ (CeO_2_) to Ce^3+^ (Ce_2_O_3_).

A literature survey revealed quite scarce data on ceria inclusion as a component in vegetable oil hydrogenation catalysts. Alouche et al. [41] studied nickel–cerium mixed oxides modified with Al. Later, the impact of different cerium amounts on the activity and *cis*/*trans* selectivity of co-precipitated NiCeAl catalysts for the deeper hydrogenation of canola oil was reported by Konkol et al. [42].

In recent studies, it has been established that metallic Ni particles were uniformly distributed on reduced CeO_2_ surfaces and stabilized over Ce^3+^ sites [43,44]. These results agree with the fundamental study of the model Ni/CeO_2_ system [45]. The authors detected a uniform distribution of metallic Ni particles on the ceria surface, and this phenomenon is explained as a result of the created surface defects as oxygen vacancies, being nucleation sites for the metallic nickel. DFT calculations applied to evaluate the metal nickel deposition on stoichiometric (Ce^4+^O_2_) and reduced (Ce^3+^_2_O_3_) cerium oxide surfaces showed that Ni is in the 2^+^-oxidation state on stoichiometric CeO_2_ and in the metallic state, Ni^0^, on reduced Ce_2_O_3_ oxide. The presented model disclosed that metallic nickel interacts with Ce^3+^ sites and creates bonds to stabilize at the surface [46,47]. This stabilization of the metallic Ni on the cerium surface determines its oxidation resistance and offers an increased number of interacting metal atoms.

An important factor that affects hydrogenation activity is the presence of an accessible and dispersed metallic Ni phase on the catalyst surface. Considering our own and other findings, we could suppose a possible improvement in the activity and selectivity of MgNi/SiO_2_ vegetable oil hydrogenation catalysts by increasing the surface metal Ni availability via modification by ceria. The proposed approach represented the introduction of CeO_2_ in two modes, namely a modifier of the SiO_2_ support and a catalyst component.

This investigation is focused on the modification of MgNi/SiO_2_ samples with CeO_2_ (1 wt.%) and aimed at affecting the uniform distribution of Ni species on a reduced CeO_2_ surface followed by stabilization on the Ce^3+^ sites. It is desirable to obtain tightly held Ce^3+^ ions supported on SiO_2_, thereby providing stabilization of the metallic Ni, as well as moderately bound Ce^3+^ ions in cases where the latter are part of the catalytic component.

The aim of the proposed work is related to the exploration and application of CeO_2_-doped catalysts to maximize the fraction of C18:1 oleic acid with the minor formation of TFAs and C18:0 stearic acid. The CeO_2_ dopant role in the studied solids was assessed by comparative analysis of the structural and surface properties, reduction and hydrogenation behavior, and FA composition attained at an iodine value of 80 during the sunflower oil hydrogenation process over ceria-doped catalysts relative to the matching undoped analogs Ni/SiO_2_ and MgNi/SiO_2_. The PXRD, SEM-EDS, and XPS combined with H_2_-TPR, H_2_-chemisoption, and HRTEM techniques for in-depth characterization were used to evaluate the precursor structure, reducibility, and surface and electronic states.

So far, no studies related to the CeO_2_-assisted design of Ni/SiO_2_ catalysts for the partial hydrogenation of vegetable oil have been reported.

## 2. Results

Commercial silica gel (SIG) was used as a SiO_2_ support to synthesize Ni-based catalyst precursors. The latter were prepared with identical weight compositions by applying two synthesis modes: (i) Ni, Mg, and CeO_2_ precipitation over bare silica gel (named Ni, MgNi, and CeMgNi) and (ii) Ni and Mg precipitation over modified silica gel with CeO_2_ (denoted as MgNi-Ce). Information that is more comprehensive is presented in Section 3, Materials and Methods.

### 2.1. Study of Catalysts Behaviour during Reduction/Activation Pretreatment

Several physicochemical methods were applied to analyze the interaction strength in the formed phyllosilicates and consider their transformation during H_2_ reduction/activation pretreatment. Characteristics of the active metallic nickel phase in the Mg- and CeO_2_-doped and undoped Ni/SiO_2_ catalyst were compared by the study of four reduced samples: Ni-red, MgNi-red, CeMgNi-red, and MgNi-Ce-red. Such detailed information could give insight into the active state of the studied catalysts regarding structure engineering with dopants such as Mg and CeO_2_ on the one hand and considering the applied approach to CeO_2_ designing of the MgNi structure by modification of the silica gel support and by the CeO_2_ addition as a component to nickel and magnesium. The provided results could be the basis for understanding the catalytic performance as the hydrogenation activity and *cis*/*trans* selectivity.

#### 2.1.1. Scanning Electron Microscopy-Energy Dispersive Spectroscopy (SEM-EDS)

**Figure 1:**
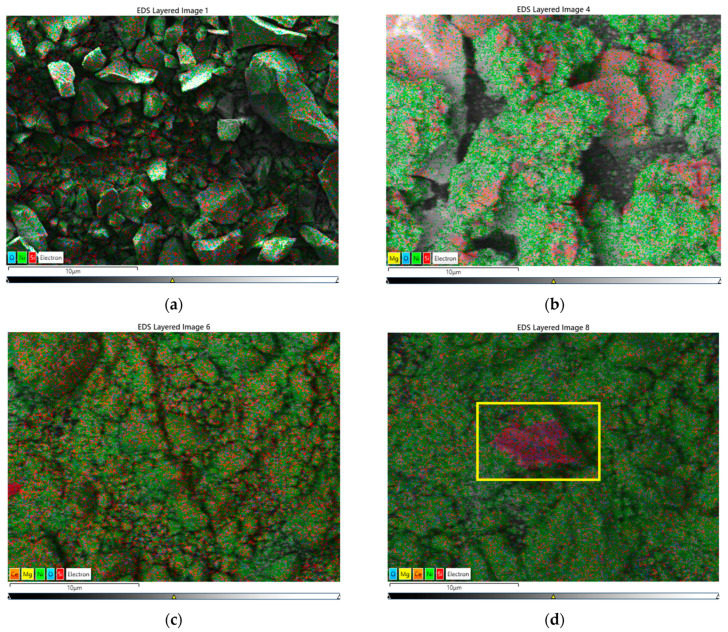
SEM images of the morphology and EDS elemental mapping at magnification 100×: (**a**) Ni-red; (**b**) MgNi-red; (**c**) CeMgNi-red; (**d**) MgNi-Ce-red.

This section presents the results of the surface microstructure and composition of the catalysts after the ex situ reduction procedure (see Section 3.4.1.) using SEM-EDS characterization. Close observation of the SEM images reveals that the morphology of the four configurations is different (Figure 1a–d). In this connection, the reduced Ni catalyst resembles a monolithic assembly of homogeneous Ni and Si components (Figure 1a). Obviously, the addition of Mg to the catalyst composition (Figure 1b) changed its topography. Redistribution due to the Mg presence makes a visible formation of flower-like clusters consisting largely of nickel species. Undoubtedly, Mg provokes surface diffusion, which changes the surface morphology of the MgNi-red catalyst. Mg doping causes concentration of the nickel species, forming dense surface assembles. On the one hand, such an effect looks to be a poor surface distribution of the nickel entities. However, on the other hand, nickel surface morphology is a precondition for an increased number of accessible active reaction sites (H_2_ and sunflower oil) and easy-going dissociation of the forming triglyceride molecules. Generally, the addition of the ceria dopant to the MgNi-red composition changed the topography, irrespective of the introduction mode, i.e., being a component of the composition (CeMgNi-red) and acting as the carrier modifier of the silica gel (MgNi-Ce-red) (Figure 1c,d), respectively. Although ceria’s weight contribution is small (1 wt.%), this causes a homogeneous distribution of nickel on the entire surface of the catalyst. The detected morphology also suggests a Ni ability during the reaction.

**Figure 2:**
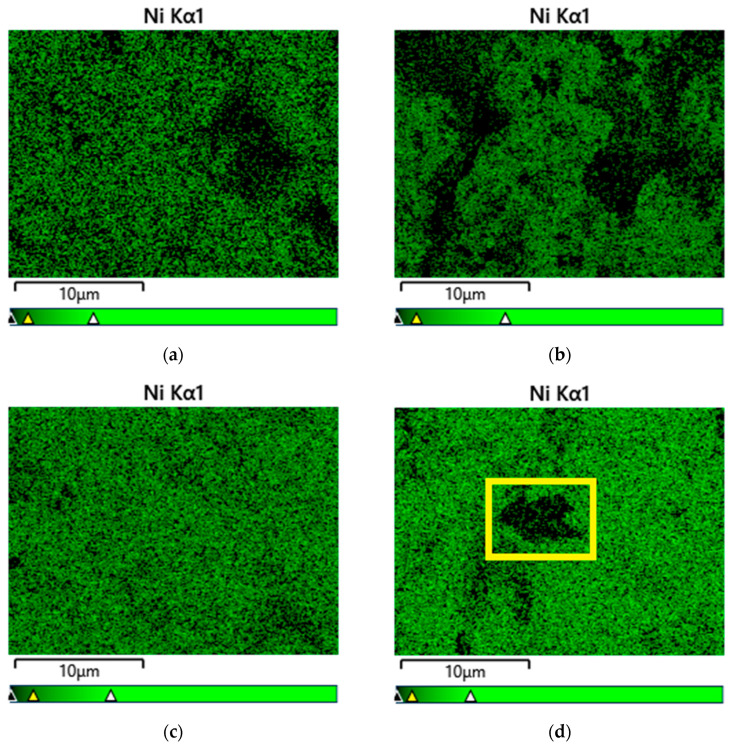
EDS elemental mapping of Ni at magnification 100×: (**a**) Ni-red; (**b**) MgNi-red; (**c**) CeMgNi-red; (**d**) MgNi-Ce-red.

Nickel microstructures of the reduced samples were examined by EDS mapping using two magnifications: the same images at magnification 100× (Figure 2a–d) and over 2000× (Figure 2a–d and Figure S1a–d). The EDS spectra clearly show a smaller size of individual Ni particles due to the magnesium impact on the reduced MgNi (Figure 2b and Figure S1b) and their surface aggregation. The ceria addition prevents aggregation, and the Ni particles are evenly distributed on the surface (Figure 2c,d and Figure S1c,d). Undoubtedly, the images show increased dispersion of the Ni microstructures upon the addition of the CeO_2_ component in the MgNi catalyst composition, which is better expressed for the co-precipitation of Ni, Mg, and Ce on silica gel with the CeMgNi catalyst (Figure 2c and Figure S1c).

The ceria-detected effect on the Ni surface microstructure has been explained as a result of surface defects (oxygen vacancies) formed during the reduction treatment being nucleation sites for the uniform distribution of Ni particles in accordance with the literature reports [45]. The fact is that CeO_2_ as a dopant may provide a solution to the enhancement of Ni distribution.

A qualitative EDS analysis of cerium dispersion is presented in Figure S2. The images display the homogenous deposition of CeO_2_ through both approaches to the introduction. However, a visible assessment indicates a larger size of the Ce microstructures if ceria is used as the modifier of the silica gel (MgNi-Ce-red).

The detected inhomogeneity of the nickel in the reduced MgNi-Ce-red catalyst outlined in yellow squares in Figure 1d, Figure 2d, and Figure S3b is due to the concentrated Si microstructures. This particular area is interpreted as more evidence for the dispersion effect of the Mg dopant on Ni distribution. It should be noted that such a place is absent in the Ce mapping image (Figure S2b). The EDS elemental mapping of Si in the MgNi-red catalyst is also of interest (Figure S3a), because it confirms this observation.

A quantitative analysis of the map sum spectra is presented. The element concentration in the four reduced catalysts is illustrated in Table 1. Compared to the theoretical composition of the undoped Ni catalyst (43.45% Ni, 20.09% Si), the data show that, after the reduction process, Ni is predominately on the catalyst surface. An evaluation of the surface-to-volume composition of the Mg- and CeO_2_-doped catalysts also confirmed the surface enrichment with Ni after reduction/activation (theoretical composition: 42.28% Ni, 1.75% Mg, 20.24% Si, and 0.81% Ce), the surface Ni/Si ratio values being above a volume Ni/Si ratio of 2.09.

In summary, the surface structure of the reduced state of the MgNi, CeMgNi, and MgNi-Ce catalysts is enriched in active Ni sites by designing the effect of Mg and Ce additives. This morphology may affect the catalytic reactivity. Moreover, Ce introduction into the composition of Ni-based vegetable oil hydrogenation catalysts could be a solution to homogenous Ni distribution.

#### 2.1.2. High-Resolution Transmission Electron Microscopy (HRTEM)

**Figure 3:**
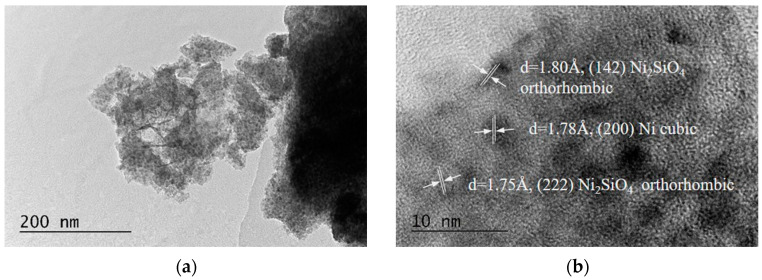

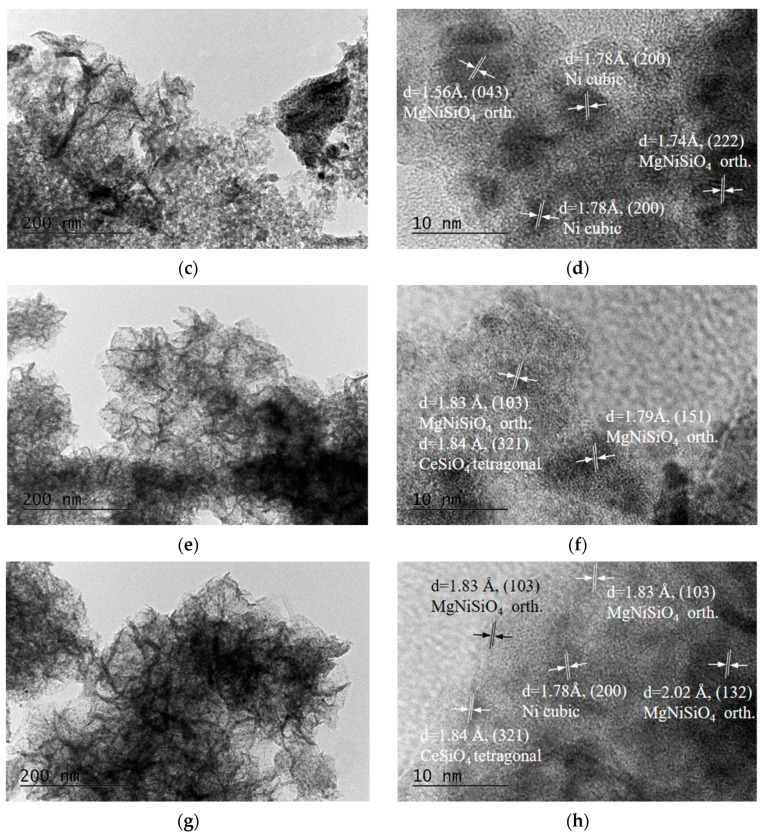
TEM images at magnification 40,000× and HRTEM images at magnification 600,000× of the reduced samples: (**a**,**b**) Ni-red; (**c**,**d**) MgNi-red; (**e**,**f**) CeMgNi-red; (**g**,**h**) MgNi-Ce-red.

Phase information and the effects of Mg and CeO_2_ doping on the microstructure of the reduced catalysts (Ni-red, MgNi-red, CeMgNi-red, and MgNi-Ce-red) were examined using transmission electron microscopy (TEM). The results are shown in Figure 3: (a,b) Ni-red, (c,d) MgNi-red, (e,f) CeMgNi-red, and (g,h) MgNi-Ce-red.

As-prepared CeO_2_-modified silica gel (CeSIG) was analyzed as a reference at magnification 40,000× (Figure S4), showing the spherical particle structure.

TEM profiles of all the reduced samples revealed the presence of spherical particles of different sizes and also the formation of flower-like structures, typical for layered materials such as phyllosilicates (Figure 3a,c,e,g). However, a comparative analysis disclosed different microstructures due to the effect of the Mg and CeO_2_ presence in the Ni/silica gel composition (Figure 3e,g). The CeMgNi-red and MgNi-Ce-red samples exhibited a network of very concentrated layer-shaped structures. Such a “branch”-like morphology of the layered phyllosilicate phase (called sheet silicates) is presented in Reference [48].

**Figure 4:**
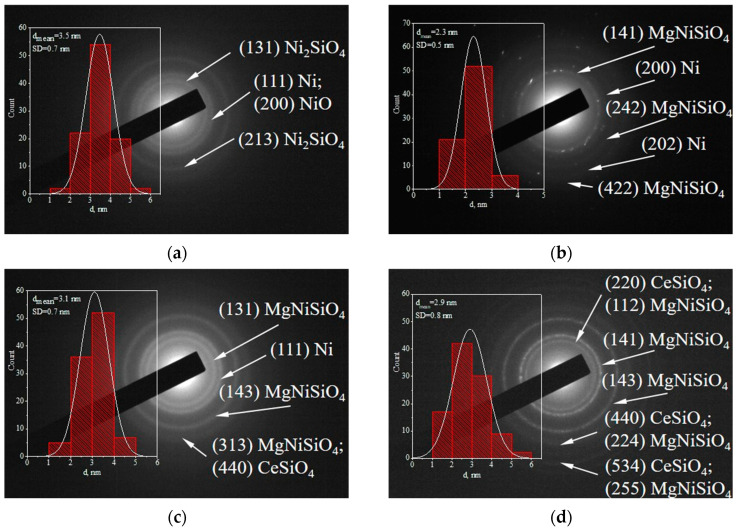
SAED patterns with insets of the Ni nanoparticle size distribution derived from HRTEM of the reduced samples: (**a**) Ni-red; (**b**) MgNi-red; (**c**) CeMgNi-red; (**d**) MgNi-Ce-red.

Information about the identified formed phases is shown in the HRTEM images (Figure 3b,d,f,h) and SAED patterns (Figure 4). The presence of cubic metallic Ni (a = 3.55200 Å, COD #96-901-3028) is registered in all the reduced catalysts. The expected silicate structures were also registered. An orthorhombic Ni_2_SiO_4_ (a = 4.77500 Å, b = 10.21600 Å, c = 5.97100 Å, COD #96-900-0634) is the reason for the layer-shaped formations in the Ni-red sample. Estimation of the interplanar distances provides information on the presence of different Ni_2_SiO_4_ phases of 1.80 Å and 1.75 Å. The magnesium introduction to Ni/SiO_2_ (MgNi-red) leads to the formation of other types of silicate structures, for example, olivine orthorhombic MgNiSiO_4_ (a = 4.73717 Å, b = 10.18700 Å, c = 5.94660 Å, COD #96-900-2615), which has two interplanar distances of 1.56 Å and 1.74 Å. The established moderately high number of layer-shaped assemblies in the CeMgNi-red and MgNi-Ce-red samples originate from the formation not only of the MgNiSiO_4_ phases but also owing to the interaction between CeO_2_ and SiO_2_ to form a silicate structure of stetindite, tetragonal CeSiO_4_ (a = 6.95640 Å, c = 6.19530 Å, COD #96-901-2498). Here, however, MgNiSiO_4_ structures are characterized by larger interplanar distances, namely 1.83 Å and 1.79 Å with CeMgNi-red and 2.02 Å and 1.83 Å with MgNi-Ce-red. These data illustrate the influence of ceria on microstructures’ defects. The presence of a free Ce-O phase was not detected.

The formed silica-supported phyllosilicates determined the co-existence of Ni, Mg, and Ce in the as-synthesized precursors on using silica gel as the SiO_2_ source. Phyllosilicate presence in the reduced samples supports the finding that its structure is hardly reducible [49]. The difference in the phase composition registered in the SAED patterns of the CeMgNi-red and MgNi-Ce-red samples (Figure 4c,d), consisting of numerous CeSiO_4_ crystallographic planes in the case of the preliminary modification of the silica gel with CeO_2_, is probably favored by the close interaction of CeO_2_ and SiO_2_ compared to the ceria co-precipitation case.

For a deeper analysis, the size distribution of the metallic Ni particles and average Ni particle size was estimated. The data are presented in the insets of Figure 4.

The engineering of the Ni/SiO_2_ composition with Mg leads to a decrease in the average metallic Ni particle size from 3.5 nm in Ni-red to 2.3 nm in MgNi-red. Undoubtedly, Mg doping inhibits Ni particle agglomeration during the reduction process. In contrast, CeO_2_ doping slightly increases the metallic Ni size to 3.1 nm in CeMgNi-red and 2.9 nm in MgNi-Ce-red. However, returning to the SEM-EDS characterization data (Figure 1 and Figure 2), EDS elemental mapping indicates a homogenous distribution of the Ni composition at the ceria presence compared to magnesium. In addition, careful analysis of the size distribution histograms also indicates differences among the four reduced samples:

- metallic Ni particle size distribution in the Ni-red sample is in the range of 1–6 nm (Figure 4a). About 55% are Ni^0^ particles 3–4 nm in size, but a considerable number of 2–3 nm and 4–5 nm sizes are also present (about 40% of a total);
- MgNi-red contains Ni^0^ particles in the frame of 1–4 nm (Figure 4b). The size distribution histogram shows a more uniform size;
- metallic Ni size distribution range increases with CeMgNi-red to 1–5 nm compared to MgNi-red (Figure 4c). The predominant formation of particles of 2–3 nm (~35%) and 3–4 nm sizes (~52%) is an additional argument;
- CeO_2_ introduction as a modifier of silica gel support in MgNi-Ce-red changes the picture of the size distribution histogram (Figure 4d). The Ni^0^ particles formed are of the sizes of 1–6 nm; however, some of them exhibit smaller sizes in contrast to CeMgNi-red, namely, 1–2 nm (~16%), 2–3 nm (~42%), and 3–4 nm (~30%). Attention should be directed to the average metallic Ni particle size being the same as CeMgNi-red, but with this kind of structure, smaller design Ni^0^ particles were formed during the reduction/activation stage.

Visibly, structural reconstruction was achieved by different approaches to the ceria introduction. HRTEM and SAED micrographs also answered whether the applied reduction condition is strong enough to decompose the observed silicate structures, which were hardly reducible. Moreover, part of the nickel species remained unreduced. The largest number of MgNiSiO_4_ and CeSiO_4_ structures existed in MgNi-Ce-red when CeO_2_ was applied as a SiO_2_ modifier in contrast with Ni and Mg co-precipitation in the CeMgNi-red sample. The registered silicate microstructures in the reduced samples could be related to the catalytic ability of the four samples. The variety in the morphological features could also be a determining factor. Given the well-known fact that the geometric surface area is an important parameter for catalyst activity, the phyllosilicate plate-like structure is expected to enhance specific surface areas, thereby increasing the number of active sites. Furthermore, smaller provided sizes of the metallic Ni particles will collaborate for more accessible active sites for surface reactions in agreement with the catalyst configuration.

#### 2.1.3. X-ray Photoelectron Spectroscopy (XPS)

The XPS technique was used to analyze the surface electronic state of the reduced catalysts. The binding energy (BE) values of the catalyst’s components are listed in Table 2. A general inspection shows higher values of the Ni2p, O1s, and Si2p photoelectrons in the spectra of MgNi-red, CeMgNi-red, and MgNi-Ce-red compared to the initial Ni-red sample. In addition, the binding energy of the Mg1s photoelectrons in CeMgNi-red and MgNi-Ce-red is also higher than that of MgNi-red. The shift to higher BEs is a result of the influence of the neighbors and confirms interactions among the components after Mg and CeO_2_ doping in accordance with the HRTEM data.

An analysis of the Ni2p peaks by curve fitting undoubtedly indicates the presence of metallic nickel on the surfaces of all the reduced samples after passivation treatment in inert Ar flow prior to sample unloading from the setup unit. The low intense energy peak (Table 2 and Figure S5a) agrees with the literature data of the Ni2p_3/2_ spectrum of the metal Ni state [50,51,52]. Ni2p_3/2_ electrons of higher BEs characterize the Ni^2+^ ions at the surface in an oxygen environment in all the reduced catalysts. The Ni^2+^ oxidation state values can be attributed to Ni ions in Ni phyllosilicate registered around 856.3 eV [48,52]. The BEs are higher with CeMgNi-red and MgNi-Ce-red, being obviously affected by the ceria presence. As the HRTEM results show, Ni^2+^ ions originate from unreduced Ni(Mg)-phyllosilicates, CeSiO_4_, and after the reoxidation of Ni^0^ during the sample mild passivation in Ar flow and transfer to analysis.

Cerium detection was not possible in the XPS spectra of CeMgNi-red and MgNi-Ce-red due to the low concentration of ceria at 1 wt% and overlap between the Ni2p_1/2_ and Ce3d_5/2_ levels. The characteristic satellite peak at about 916 eV in the Ce3d regions, being the fingerprint of the Ce^4+^ oxidation state [43,53], is correspondingly absent.

The reduction degree is displayed by comparison between the peak areas of the Ni^2+^ oxidation state and Ni^0^ metallic state via the Ni^2+^/Ni^0^ ratios in Table 2. The MgNi-red lowest value of 2.3 indicates that the degree of reduction is increased by Mg doping, and the rest of the MgNiSiO_4_ phyllosilicate is less. The CeO_2_ doping synthesis approach did not affect the reduction degree of CeMgNi-red related to Ni-red and noticeably increased upon usage of CeO_2_ as the SiO_2_ support modifier. However, because XPS is a surface sensitive technique, the Ni^2+^/Ni^0^ ratios also reflect the oxidation degree of the surface nickel. Therefore, excessive Ni^0^ reoxidation by existing CeO_x_ species cannot be excluded, bearing in mind ceria’s great oxygen storage capacity.

Table 3 lists the results of the quantitative XPS analysis. Another important factor is nickel dispersion after reduction/activation as estimated by calculation of the Ni/Si ratio. Nickel availability on the surface after reduction treatment was improved by magnesium in the MgNi-red catalyst composition. Similarly, this effect exists with MgNi-Ce-red relative to Ni-red.

To sum up, engineering of the surface electronic properties is also achievable for reduced catalysts. BE shifts indicated interactions among the components and silica because of the formation of phyllosilicates. This change in the electron cloud density of the metallic Ni combined with its amount at the active surface could impact the hydrogenation performance and *cis*/*trans* selectivity according to the catalyst composition and preparation.

#### 2.1.4. Powder X-ray Diffraction (PXRD)

**Figure 5:**
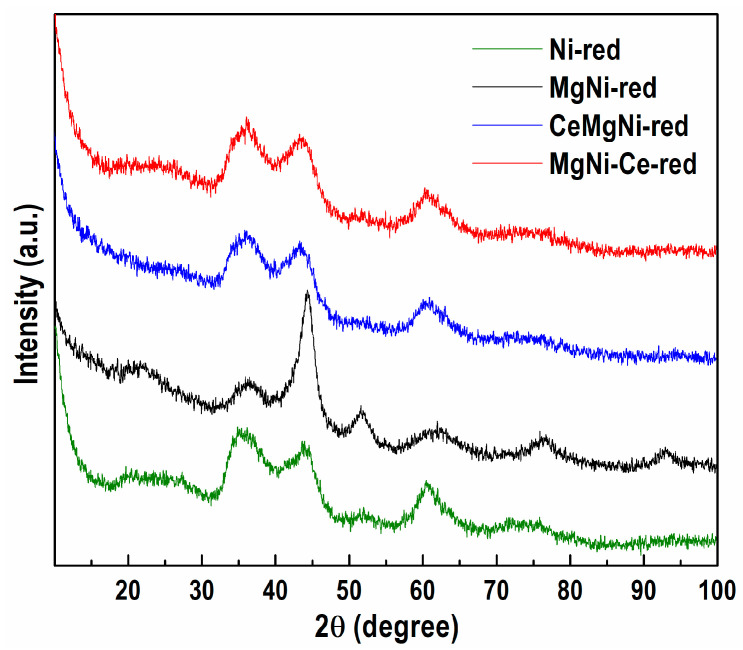
PXRD patterns of the ex situ reduced samples.

The registered peaks at ≈34–35° and ≈60° in the PXRD patterns of all the reduced precursors (Figure 5) are ascribed to the presence of nickel phyllosilicate phases of pecoraite (Ni_3_Si_2_O_5_(OH)_4_, ICDD-PDF file 00-049-1859) and nepouite (Ni, Mg)_3_Si_2_O_5_(OH)_4_, ICDD-PDF file 00-025-0524). The result obtained agrees with the PXRD analysis of the as-synthesized samples, which indicates the formation of the Ni silicate phases (Figure S6). This finding is related to the Ni^2+^ species that strongly interacts with silica through Ni-O-Si bonds in the Ni phyllosilicate, which remained after reduction. The aforementioned stronger interaction needs a higher reduction temperature. PXRD analyses showed that the Ni phyllosilicate species were not completely reduced under the applied reduction conditions.

Generally, silica-supported Ni phyllosilicate is partially reduced in H_2_ atmosphere, and therefore, the Ni-silica interface still contains ionic Ni species, though of low concentration. Some of the Ni^2+^ ions are reduced to metallic particles, while the remaining part is retained in the Ni phyllosilicate phase. Bound strongly to the silica surface, these unreduced Ni species act as anchoring sites for the metallic Ni particles, leading to their stabilization, favoring metal Ni dispersion, and sintering resistance. Consequently, the unreduced phyllosilicate structure acted as support for the catalytically active Ni^0^ particles. Silica plays the role of spacer of the nickel species Ni^2+^ and Ni^0^, thus preventing their sintering [54,55,56]. Finally, metallic nickel obtained by the reduction of Ni phyllosilicate has a fine size at a high nickel content and high dispersion.

While the HRTEM analysis revealed the presence of a metallic Ni phase in all the reduced samples, the PXRD technique registered metallic Ni only with MgNi-red. The recorded reflections at 2*θ* = 44.5, 51.7, 76.4, and 92.9° (Figure 5), which are typical of the metallic nickel phase in cubic symmetry (ICDD-PDF file 00-004-0850), indicate the co-existence of Ni phyllosilicate and Ni metal in the reduced MgNi-red sample.

The broadening of the metallic nickel diffraction lines signifies small metallic nickel crystallites. The poor crystallinity of the MgNi-red sample predetermines that an evaluation of the mean Ni^0^ crystallite size is practically impossible.

Additionally, the registered high background below 2*θ* ≈ 15° indicates advanced amorphization of the observed phases.

### 2.2. Temperature-Programmed Reduction with Hydrogen (H_2_-TPR)

Activation of the vegetable oil hydrogenation catalysts is commonly accomplished by reduction of the nickel species present at the silica surface to metallic nickel to obtain the maximum amount of nickel surface area related to the catalyst activity.

Temperature-programmed reduction with hydrogen (H_2_-TPR) is able to provide detailed information on Ni^2+^ reducibility and characterize the interaction between metallic Ni species and SIG-based supports.

Two modes were performed to reveal the precursor redox properties: normal/typical TPR from 50 to 900 °C and isothermal reduction of the samples at 430 °C and 490 °C for 120 min. The isothermal reduction was performed to gain a more comprehensive view of the H_2_-TPR results and explain the hydrogenation activity of the catalysts studied. The results obtained were compared with Ni and MgNi catalysts to clarify the ceria doping effect.

**Figure 6:**
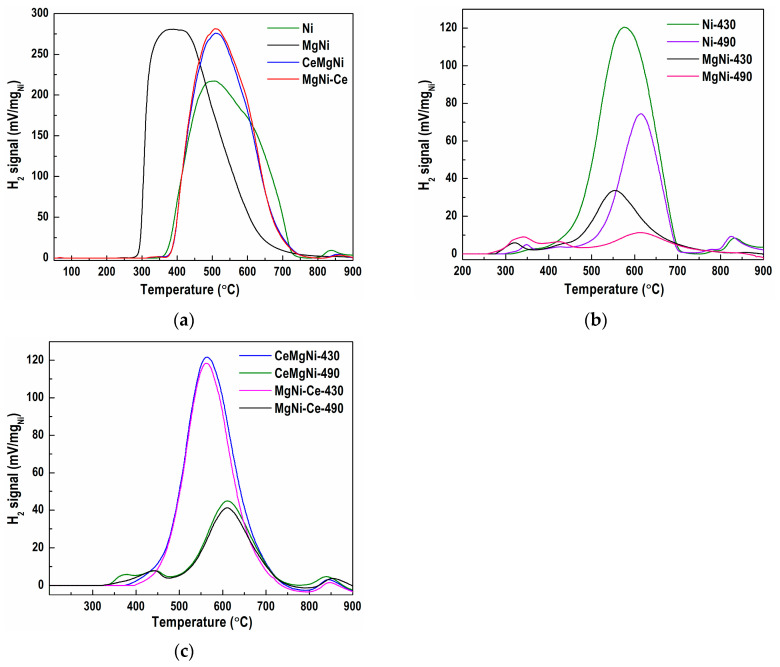
H_2_-TPR profiles of (**a**) all precursors in the range 50–900 °C, (**b**) Ni and MgNi precursors after isothermal reduction at 430 and 490 °C, and (**c**) CeMgNi and MgNi-Ce precursors after isothermal reduction at 430 and 490 °C.

Comparison of the TPR profiles of the four samples in the range 50–900 °C (Figure 6a) displays differences in shape, reduction temperature, and hydrogen consumption values, thus revealing quite different reduction behavior of the formed Ni species. The return to the baseline of the TPR curves is a good criterion for complete reduction of the nickel species at 900 °C. Multiple reduction peaks with poorly resolved maxima characterize precursor TPR profiles, indicating a complex interaction among the components. The reduction process in precursor Ni starts at ≈315 °C (Figure 6a). The profile exhibits a broad asymmetrical signal between 350 and 750 °C, a main temperature maximum (T_max_) at 500 °C with a shoulder around 650 °C, and a broad small intensity peak at 838 °C. This suggests a variety of different Ni species formed through the synthesis procedure. Isothermal reduction at 430 °C (Figure 6b) indicates a notable amount of unreduced nickel species, namely via a low-temperature shoulder at 366 °C, a T_max_ peak at 577 °C constituting 35.8% of the total TPR (Figure 6a), a high-temperature shoulder at 782 °C, and small peak at 832 °C. Isothermal reduction of the Ni precursor at 430 °C (Figure 6b) indicates a notable amount of unreduced nickel species, namely via a low-temperature shoulder at 366 °C, a T_max_ peak at 577 °C constituting 35.8% of the total TPR (Figure 6a), a high-temperature shoulder at 782 °C, and a small peak at 832 °C. After isothermal reduction of the Ni precursor at 490 °C (Figure 6b), the profile shows a small low-temperature peak at 347 °C and a shoulder at 425 °C.

Further, the T_max_ peak at 615 °C still covers 16.9% of the total TPR (Figure 6a), accompanied by a high-temperature shoulder at 777 °C and a small peak at 825 °C. While reduction at 430 and 490 °C causes the occurrence of low- and high-temperature shoulders which positions are slightly moved towards lower temperatures, the main temperature maxima are shifted to higher temperatures. It should be pointed out that a considerable amount of unreduced nickel species is present in the reduced Ni precursor arising from the reduction of nickel phyllosilicate. It is possible that a higher reduction temperature could cause agglomeration of the metallic nickel particles, which could result in a lower catalytic activity during the process of sunflower oil hydrogenation.

Magnesium introduction to the Ni precursor, sample MgNi, considerably changed the overall TPR profile (Figure 6a). A sudden increase in the hydrogen consumption at the beginning of the reduction at a temperature of about 250 °C, a fast reach to the maximum temperature, T_max_ of 390 °C, which is significantly lower than that of the Ni precursor, and heavy asymmetry of the TPR profile exhibit a strong tailing of the signal above 420 °C. All these suggest the presence of the prevailing species reducible at lower temperatures in the MgNi precursor. No high-temperature shoulders or peaks were detected. This indicates that Mg promotes reduction at lower temperatures, as evident in the TPR profiles after isothermal reduction at 430 and 490 °C (Figure 6b). At 430 °C, almost the entire precursor was reduced with a low-temperature peak at 320 °C and shoulder at 418 °C and T_max_ peak of 555 °C that covered 8.6% of the total TPR profile surface. Reduction at 490 °C further diminished the number of unreduced species to only 4.6% of the total TPR profile.

The aforementioned results show that the NiMg phyllosilicates from the MgNi precursor were nearly completely reduced at a temperature of 430 °C, and therefore, MgNi-red is expected to exhibit a significantly higher catalytic activity. The increase in reducibility also signified a descending profile of the metal–support interaction, in which weakly bonded nickel phases to the support would be more readily reduced to metallic Ni^0^.

The two precursors prepared by the modification of MgNi with CeO_2_ (CeMgNi and MgNi-Ce) were also subjected to TPR measurements. Surprisingly, both TPR profiles (Figure 6a) show significantly different signals from those of the MgNi precursor, being almost identical to the profile of the Ni precursor and somewhat narrower, which means reduction of a more homogeneous nickel phase, as detected by SEM investigations of the reduced precursors (See Section 2.1.1). The profiles exhibit asymmetric peaks with close hydrogen consumption values. The similarity of the TPR curves is noticeable in the range from 350 to 900 °C with a peak maximum at about 510 °C and very slightly pronounced high-temperature shoulders at 857 and 861 °C with CeMgNi and MgNi-Ce, respectively. TPR analysis indicates that the ceria introduction hindered the precursor reduction by shifting to higher temperatures. The reduction of CeMgNi and MgNi-Ce at 430 °C (Figure 6c) caused a slight shift of the T_max_ peak to a lower temperature by about 15 °C relative to that of the Ni precursor covering 30.9% (CeMgNi) and 27.2% (MgNi-Ce) of the total TPR profiles. A minor shift to a higher temperature of the shoulders at ≈850 °C by about 14 °C in comparison with the Ni precursor was also observed for the Ce-containing samples. An increase in the reduction temperature to 490 °C (Figure 6c) improved the reduction of the CeMgNi and MgNi-Ce precursors. The TPR profiles displayed low-temperature shoulders at 373 and 443 °C with CeMgNi and also at 443 °C for MgNi-Ce. The same T_max_ at 611 °C was registered with both precursors, comprising 12.0% (CeMgNi) and 10.3% (MgNi-Ce) of the total TPR profiles. High-temperature shoulders at 839 °C (CeMgNi) and 854 °C continued to be present in both TPR profiles. This reduction behavior is explained not only by the presence of hardly reducible nickel phyllosilicate structures but also by the existence of a large number of MgNiSiO_4_ and CeSiO_4_ structures in MgNi-Ce-red, as was clarified by the SEM study of the reduced precursors (See Section 2.1.1). The formation of nickel cerates should not be overlooked either.

The comparison of the H_2_-TPR profiles of the investigated precursors after isothermal reduction at 430 °C and 490 °C is presented in Figure S7a,b, respectively.

The H_2_-TPR studies revealed different types of nickel species, which are associated with the degree of interaction with the silica support. While the peaks below 450 °C are due to weakly bonded nickel species to the support, the peaks above 500 °C are ascribed to Ni species having strong interactions with the silica. That is why different amounts of hardly reducible silicate structures are still present in the reduced precursor at 430 and 490 °C because of strong interactions between nickel species and the silica support, thus forming silicates.

### 2.3. H_2_ Chemisorption

H_2_ chemisorption was applied as the most employed technique for determination of the essential parameters for nickel-based catalysts such as metallic Ni-specific surface area (SSA_Ni_) and metallic Ni dispersion (D_N**i**_), as well as Ni particle size. The provided data are given in Table 4. The results of the specific nickel surface area are expressed per gram of nickel. Additionally, Table 4 includes calculated values of the Ni particle size for each catalyst, as determined for two assumed particle shape models: cubic and hemispherical.

Clearly, the MgNi-red catalyst has the highest values of SSA_Ni_ and D_Ni_ and smallest Ni particle size among all the catalysts used, while the Ni-red catalyst has the lowest values of SSA_Ni_ and D_Ni_ and highest Ni size. These data, in combination with the previously presented surface analyses by SEM-EDS and XPS, undoubtedly confirm a higher metallic Ni accessibility in Mg-doped Ni/SiO_2_. The cerium role as a dopant also improves the surface features of metallic nickel.

It is important to emphasize again that Mg and Ce implementation provides design reconstruction, thus increasing the Ni^0^ adsorption sites for hydrogen reagent molecules.

### 2.4. Performance of the Catalysts

#### 2.4.1. Partial Hydrogenation of Sunflower Oil

**Figure 7:**
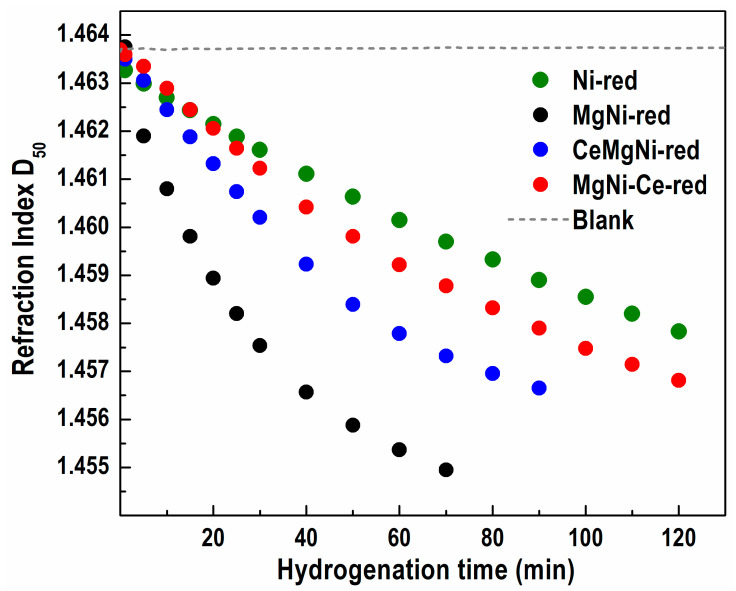
Sunflower oil hydrogenation activity vs. reaction time of the studied catalysts.

Catalysts reduced at 430 °C for 5 h and impregnated with paraffin oil were subjected to activity tests by the partial catalytic hydrogenation of sunflower oil. The hydrogenation efficiency was monitored by detecting changes in the refractive index (RI) at 50 °C of the starting oil and periodically collected partially hydrogenated oils with time. the initial sunflower oil had a RI of 1.46368. RI descending values as a function of hydrogenation time for the studied catalysts are given in Figure 7.

As anticipated, the RI values for all catalysts decreased as the FA molecules were progressively saturated during hydrogenation. Furthermore, the graph gradient signified a RI decline rate, whereby a sharper gradient represented an increase in hydrogenation activity. Among the synthesized catalysts, the MgNi-red sample using undoped SIG support manifested the highest activity by attaining a final RI value of 1.45495 after a reaction time of 70 min.

Co-precipitation of Ce, Mg, and Ni on undoped SIG support (CeMgNi-red catalyst) produced a RI value drop to 1.45665 in 90 min. By means of CeO2-doped SIG support, MgNi-Ce-red, the less active catalyst, demonstrated a decrease in RI level to 1.45681 after 120 min. As the least active, the Mg-free Ni-red catalyst employing undoped SIG support reached a RI value of 1.45783, also within 120 min.

The collected data revealed that the catalysts hydrogenated sunflower oil to the same RI value (same conversion) for different reaction times (different activity). In other words, higher RI values and longer reaction times of the ceria-doped catalysts compared to the MgNi-red and Ni-red undoped catalysts indicate a lower hydrogenation activity of the former entities.

The hydrogenation activity of the studied catalysts was confirmed by TPR examinations. They revealed different amounts of reduced Ni phyllosilicates at 430 °C. This finding is related to the diverse types of nickel species with varied degrees of interaction with silica support. The significantly higher hydrogenation activity of the MgNi-red catalyst is a result of weakly bonded nickel species to the support, which are practically completely reduced at 430 °C. A lower activity of the other reduced catalysts is explained by presence of hardly reducible silicate structures at 430 °C because of strong interactions between nickel species and the silica. It should be pointed out that a considerable number of unreduced nickel species occurred in the Ni-red catalyst, thus showing the lowest hydrogenation activity.

#### 2.4.2. Selectivity and Composition of the Hydrogenated Products

In the vegetable oil industry, changes in the iodine value (IV; grams of iodine that are absorbed by 100 g of oil) are an important factor in quality control. It is often employed to target fractionation processes to the desired degree of hydrogenation to obtain the required amount of product under the optimum operating conditions [57]. Currently, the most commonly applied method for IV determination is based on calculation of the concentration of fatty acid methyl esters obtained by gas chromatography (GC) analysis represented as the molar or mass fraction [58].

**Figure 8:**
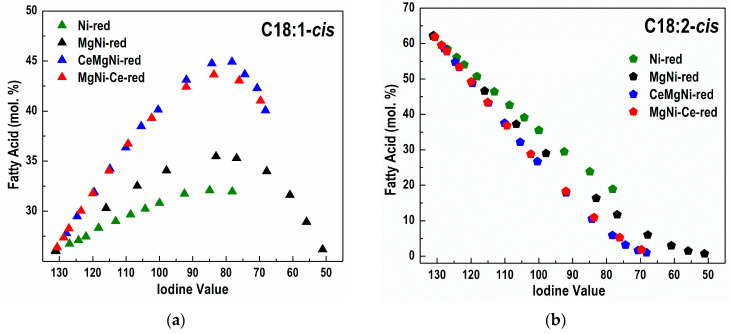

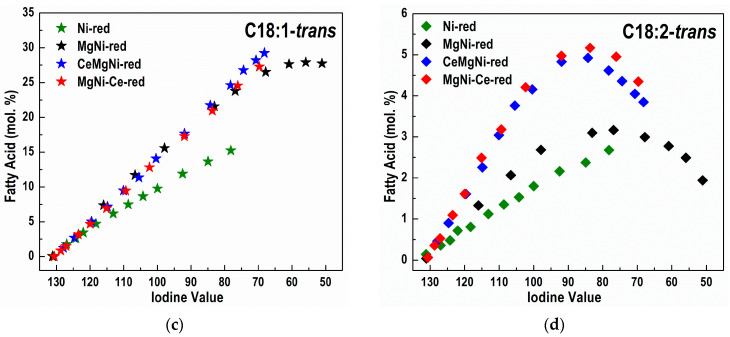
Change in the composition of (**a**) C18:1-*cis*, (**b**) C18:2-*cis*, (**c**) C18:1-*trans*, and (**d**) C18:2-*trans* FAs vs. IV during partial sunflower oil hydrogenation using different catalysts.

Differences in the catalyst activity reflect on different FA profiles obtained during the partial hydrogenation of sunflower oil and different FAs compositions (Figure S8 and Figure 8a–d, Table 5). Concomitant with hydrogenation, a *cis*/*trans* isomerization reaction takes place.

It is important to note that the MgNi-red catalyst demonstrated the highest hydrogenation activity but is not selective enough with respect to the formation of C18:1-*cis* and C18:0 FAs (Table 5).

The positive effect of CeO_2_ presence in the CeMgNi-red and MgNi-Ce-red catalysts is clearly seen when converted amounts of the fatty acids during hydrogenation are compared at IV = 80 (Table 5). Ceria existence in CeMgNi-red and MgNi-Ce-red had the following impacts:

- inhibited formation of C18:0 stearic acid;
- increased amount of C18:1, especially C18:1-*cis*;
- diminished amount of C18:2, especially C18:2-*cis*;

Both Ce-containing catalysts are more selective than Ni-red and MgNi-red in relation to the amounts of C18:0, C18:1, C18:2, C18:1-*cis,* and C18:2-*cis* FAs in the hydrogenated products.

The Ni-red catalyst shows the smallest amount of formed C18:1-*trans* and C18:2-*trans* FAs (Figure 8). However, this catalyst exhibited the lowest hydrogenated activity. The non-selective Ni-red catalyst yields, at the same IV, a partially hydrogenated oil with both more saturated C18:0 FA and more non-hydrogenated molecules than the other catalysts. As fully hydrogenated triglyceride molecules have no *cis* or *trans* isomers, and since the double bonds in molecules untouched by the catalyst are still in the *cis* configuration, the non-selective catalyst produces less *trans* FAs.

The rest of the catalysts demonstrated comparable selectivity in terms of C18:1-*trans* and C18:2-*trans* FA formation with MgNi-red (Table 5).

An attempt was also made to enhance the hydrogenation activity of the CeO_2_-doped catalysts by raising the reduction temperature from 430 to 490 °C. However, increasing the reduction temperature by 60 °C diminished the activity of both catalysts. A comparison of the activity at the two reduction temperatures follows the order

MgNi-430 > CeMgNi-430 > MgNi-Ce-430 ≅ CeMgNi-490 > MgNi-Ce-490 > Ni-430

In addition, the MgNi-Ce catalyst, reduced at 490 °C, needed a longer hydrogenation time of 150 min than the sample reduced at 430 °C.

Although the activity of ceria-doped catalysts was lower relative to MgNi-red, these catalysts, especially CeMgNi-red, were significantly more active than Ni-red. Additionally, CeO_2_ modification enabled the design of C18:1 selective catalysts compared to the Mg-doped nickel catalyst (MgNi-red) and undoped nickel catalyst (Ni-red). Therefore, CeO_2_ could be used to design a C18:1 selective nickel catalyst that, within a reasonable hydrogenation period, might reduce the iodine number by about 50 units.

The analysis of the potential selectivity of the catalysts, observed through the difference in concentration (Δ) of a specific species (e.g., C18:1-*cis*) in the partially hydrogenated sample compared to the initial concentration of that species (C18:1-*cis*-Int) in the starting, non-hydrogenated oil, can serve as a good indicator of selectivity. For different catalysts, along with the increase in the content of *cis* isomers during hydrogenation, the content of *trans* isomers also increases, as illustrated in Figure 8a,c.

A more accurate approach to estimate the selectivity of different catalysts towards the cis/trans isomers of C18:1 in partially hydrogenated products can be achieved by comparing the ratio of absolute changes in the concentrations of *cis* (ΔC18:1-*cis*) and *trans* (ΔC18:1-*trans*) fatty acids (e.g., ΔC18:1-*cis*/ΔC18:1-*trans*) for a selected iodine value.

The values in the second column of Table 6, related to the ceria-modified catalysts, are about 4.7 times higher than the values for the Ni-red catalyst and are more than 20% higher than the values for the MgNi-red catalyst. This confirms that the CeO_2_ modification is an effective approach for achieving C18:1-*cis* selectivity. The observed difference between CeO_2_-modified catalysts and MgNi-red catalysts is due to the similar changes in the content of C18:1-*trans* (approx. 23% for all three catalysts) and a significantly higher absolute increase in the content of C18:1-*cis* in the ceria-modified catalysts (Table 5).

In analyzing the overall selectivity for C18:1-*cis*, it is crucial to consider the contribution of all *trans* species present in the partially hydrogenated oil sample. The accumulation of 18:2-*trans* species can distort the *cis*-selectivity values, negating the positive effects generated by a high C18:1-*cis* content. The ratio of the change of 18:1-*cis* species to the total change of all trace fatty acids present in the samples of partially hydrogenated oils, for all catalysts, is provided in the third column of Table 6. The difference in the relative ratios between the CeO_2_-modified catalysts and MgNi-red catalysts, although slightly smaller, remains significant (1.59 vs. 1.38), confirming the selectivity of the CeO_2_-modified catalysts.

### 2.5. Catalysts Reduced Structure—Catalysts Vegetable Oil Hydrogenation Activity—Cis/Trans Selectivity Interfaces

**Figure 9:**
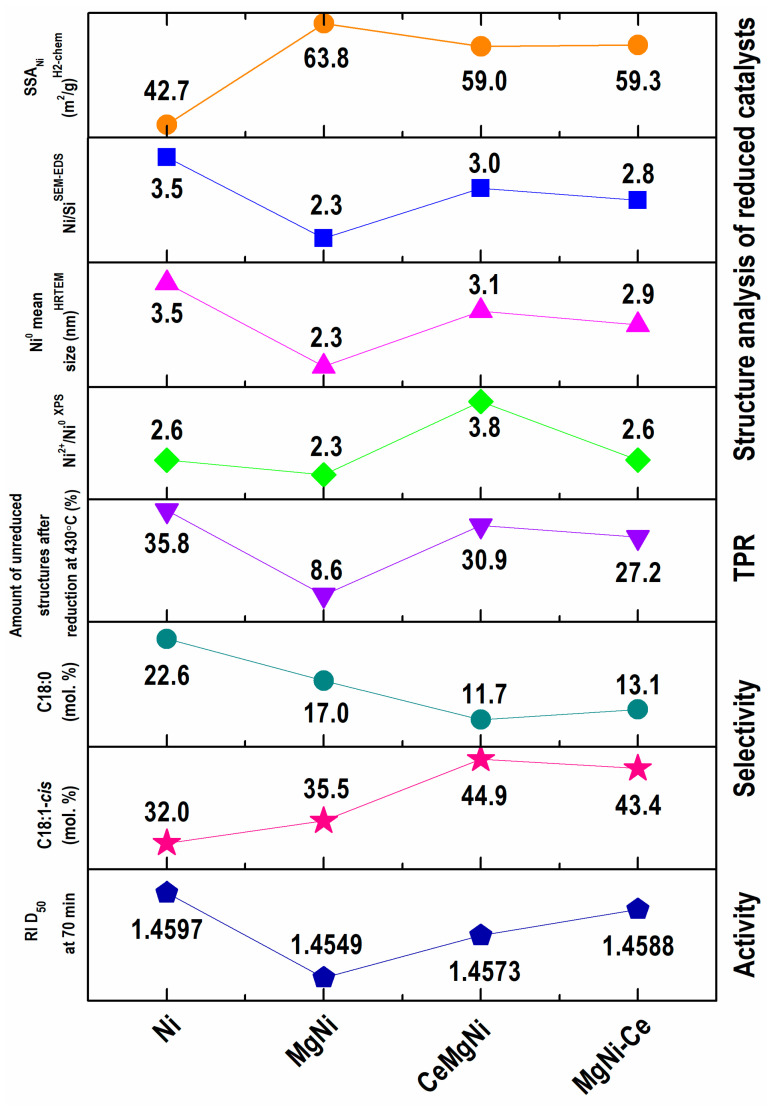
Comparison among the selected data from the characterization of the reduced samples.

The applied strategy toward surface and bulk structure engineering via CeO_2_ introduction to the MgNi configuration was aimed at metallic nickel phase distribution in reduced catalysts and accessibility to the vegetable oil hydrogenation reaction. The expectation was to achieve enhanced Ni/silica-based catalytic performance and improved *cis*/*trans* selectivity. Figure 9 summarizes the physicochemical properties and catalytic performance of the catalysts.

Cooperating SEM-EDS, HRTEM, XPS spectroscopy and H_2_ chemisorption data reveal the reconstruction role of magnesium and ceria for the morphology, microstructure, electronic properties, specific surface area of nickel, and metallic Ni particle size and dispersion in the active state of the Ni/SiO_2_ configuration. It is clearly seen that magnesium provides a more metallic nickel phase of smaller average particle sizes on the catalyst surface, thus being accessible for hydrogen and big triglyceride molecule reagents, and this structure designing reflects in a higher activity of the MgNi composition (Figure 9). The results obtained for specific surface areas of nickel were correlated with other surface analyses by SEM-EDS and XPS and undoubtedly confirmed a higher metallic Ni accessibility for most active Mg-doped Ni/SiO2 catalysts. As well, the calculated values of the Ni particle size by H_2_ chemisorption (Table 4) are in accordance with those calculated by the HRTEM bulk structure analysis (Figure 9).

Exploration of ceria designing of the MgNi structure using two modes: (i) modification of the silica gel support and (ii) CeO_2_ introduction to Ni and Mg as a third component also illustrates structure engineering via the increased homogenous deposition of surface nickel in reduced CeMgNi and MgNi-Ce precursors. Both configurations exhibit similar structural features, as briefly plotted in Figure 9.

The average Ni^0^ particle size is higher relative to a reduced MgNi precursor. Obviously, these data are due to the numerous sizes of the Ni^0^ particles in the range of 1–6 nm in contrast to a more uniform Ni^0^ size of 1–4 nm in the MgNi precursor. In addition, the provided surface reconstruction leads to higher *cis*/*trans* selectivity, which is better pronounced with the CeMgNi catalyst configuration. This fact clearly confirms a positive application of ceria as the catalyst dopant to maximize the C18:1-*cis* oleic acid fraction accompanied by the minimal formation of *trans*-FAs and C18:0 stearic acid. The observed hydrogenation behavior of the CeMgNi and MgNi-Ce catalysts can be explained by the possible reoxidation of active metallic Ni particles during the reaction owing to non-intimate Ce-Ni contact disturbed by the Mg presence. Because of ceria’s great oxygen storage capacity and possible existence of free CeO_x_ species, the catalysts may readily lose activity. This fact points to further search for a solution to achieve higher hydrogenation activity by improving the dopant balance in the catalyst composition. This issue could offer the most stable Ni^0^ particles through optimal bonding to Ce^3+^ sites formed by lattice oxygen transfer during reduction/activation of the precursors. It is well known that, under reduction conditions, this bond is created according to the equation Ni^2+^–CeO_2_–Ni^0^−CeO_2−x_ [59].

The important highlight is designing the reconstruction of the structure and surface of Ni/SiO_2_ catalysts via Mg and CeO_2_ doping aimed at increasing the adsorption metallic nickel sites to the hydrogen reagent molecules and improvement of the hydrogenation activity and *cis*/*trans* selectivity.

## 3. Materials and Methods

### 3.1. Materials and Reagents

Commercial silica gel (SIG) of the mesoporous type with 0.8–1.0 mm particle size after grinding was used as a SiO_2_ support for sample synthesis. SIG was kindly provided by ALUSIL Ltd. (Sofia, Bulgaria).

Reagents used in this work: nickel nitrate hexahydrate, Ni(NO_3_)_2_·6H_2_O, magnesium nitrate hexahydrate, Mg(NO_3_)_2_·6H_2_O, cerium nitrate hexahydrate, Ce(NO_3_)_3_·6H_2_O, and sodium carbonate anhydrous, Na_2_CO_3_, were of “pro-analyze” purity grade, supplied by Sigma-Aldrich (Steinheim, Germany), and utilized without further purification. Fresh distilled water was used to prepare the necessary solutions.

Refined sunflower oil (“Vital”, Vrbas, Serbia) was employed as the feed for the hydrogenation reaction. Fatty acid content of the starting sunflower oil determined by gas chromatography was as follows (mol %): C16:0 = 7.2; C18:0 = 4.0; C18:1-*cis* = 26.0; C18:1-*trans* = 0.02; C18:2-*cis* = 62.2; C18:2-*trans* = 0.06; C18:3-*cis* = 0.10; C20:0 = 0.22; C22:0 = 0.20.

### 3.2. Sample Preparation

#### 3.2.1. Modification of SIG Support by Ceria

Modification of SIG with 1 wt.% CeO_2_ was accomplished by ceria direct deposition on SIG suspended in distilled water as based on Ce(NO_3_)_3_·6H_2_O precipitation with 0.9M Na_2_CO_3_ at constant pH = 10 and a temperature of 60 °C. After filtering and washing with distilled water to complete NO_3_^−^ ion removal by testing with a solution of diphenylamine in H_2_SO_4_, the modified support was dried at 120 °C for 20 h. Subsequently, the solid was calcined at 400 °C for 2 h, aiming to achieve the formation of cubic ceria. CeO_2_-doped silica gel was denoted as CeSIG. A ceria concentration of 1 wt.% was selected as appropriate to exclude the possible reoxidation of the further synthesized Ni-based catalyst under operating conditions due to ceria’s well-known high capacity to store and release oxygen by Ce^3+^/Ce^4+^ surface sites related to a rapid interchange between the Ce^3+^ and Ce^4+^ redox couple.

#### 3.2.2. Synthesis of the Precursors

Precursors were prepared with identical compositions, as SiO_2_/Ni = 1.0 and Mg/Ni = 0.1, and by the same synthesis route regardless of the type of the SIG-based support. The procedure involved co-precipitation of 0.5M nitrate solution with 0.9M Na_2_CO_3_ solution in two ways:

(i) on unmodified SIG (Ni/SIG, MgNi/SIG, and CeMgNi/SIG;
(ii) on CeO_2_-doped SIG (MgNi/CeSIG).

A definite volume of distilled water containing suspended support was placed in a five-necked glass reactor equipped with a stirrer, pH electrode, thermocouple, and reflux condenser. The suspension was heated up to 90 °C, and a pH level of 10.0 ± 0.2 was adjusted with 0.9M Na_2_CO_3_ solution. Then, proper amounts of nitrate and Na_2_CO_3_ solutions were simultaneously introduced to the reactor by means of two peristaltic pumps with a reactant feed flow rate of 1 L h^−1^ under stirring at 265 rpm. The resulting slurry was aged for 60 min in the mother liquor under the above conditions. It was then filtered and washed thoroughly with hot distilled water until the pH of the filtrate decreased to ≈7 and there was an absence of NO_3_^−^ ions.

All precipitates such as Ni/SIG, MgNi/SIG, CeMgNi/SIG, and MgNi/CeSIG were dried at 120 °C for 20 h and denoted in the text and figures as Ni, MgNi, CeMgNi, and MgNi-Ce, respectively, for convenience.

The theoretical composition of the undoped Ni catalyst is 43.45% Ni and 20.09% Si and that of the Mg-doped catalysts is 42.28% Ni, 1.75% Mg, and 20.24% Si. The CeO_2_ concentration matches 0.81% Ce. The actual sample composition measured by X-ray fluorescence spectrometry (XRF) indicated the accuracy of the synthesis approach (see Table S1 in the Supplementary Materials).

### 3.3. Sample Characterization Methods

#### 3.3.1. X-ray Fluorescence Spectrometry (XRF)

Sample compositions of the precursors were determined by X-ray fluorescence measurements applying energy-dispersive X-ray fluorescence analyzer ED-XRF SPECTROSCOUT, XRF Analyzer Pro software v. 3.6, SPECTRO AMETEK Analytical Instruments GmbH (Kleve, Germany). SPECTRO XRF Analyzer iCAL (Intelligent Calibration Logic) Data Manager SPECTRO AMETEK Analytical Instruments GmbH (Kleve, Germany). https://extranet.spectro.com/-/media/821555D7-08F3-4DD0-82E3-118899DC01DF.pdf (accessed on 28 May 2024).

#### 3.3.2. SEM-EDX Measurements

SEM-EDS measurements of the reduced catalysts were performed under conditions of tungsten filament applying a resolution of 3.5 nm at 30 kV, accelerating voltage of 200 V, and spectroscopic resolution at Mn-Kα and 1 kcps 126 eV. The EDS detector was a Bruker-Quantax 200 (Tokyo, Japan). The EDS software (https://nano.oxinst.com/products/aztec/aztecone accessed on 28 May 2024) used was AZtecOne of Oxford Instruments.

#### 3.3.3. High-Resolution Transmission Electron Microscopy (HRTEM)

The sample phase composition was determined by registered selected area electron diffraction (SAED) patterns and high-resolution transmission electron microscopy (HRTEM) images. Measurements were carried out to gain further insight into existing phases in the reduced catalysts using a transmission electron microscope Jeol JEM 2100 (JEOL, Tokyo, Japan) at 200 kV accelerating voltage in the conventional mode. Phase identification was performed by means of Match software 3.13 Version (Crystal Impact, Bonn, Germany) and Crystallographic Open Database (COD).

#### 3.3.4. X-ray Photoelectron Spectroscopy (XPS)

The XPS studies were performed in an ESCALAB MK II system (VG Scientific Ltd., now Thermo Fisher Scientific Ltd., East Grinstead, UK) using Al Kα radiation with an energy of 1486.6 eV. The pressure in the chamber was 1.10^−8^ Pa. The C1s line of adventitious carbon at 285.0 eV was used as the internal standard to calibrate the binding energies. The photoelectron spectra were corrected by subtracting a Shirley-type background and were quantified using the peak area and Scofield’s photoionization cross-section. The accuracy of the binding energy measured was ±0.2 eV.

#### 3.3.5. Powder X-ray Diffraction (PXRD)

The powder X-ray diffraction (PXRD) technique was used to determine the phase composition of the as-synthesized and reduced precursors. The patterns were conducted at room temperature within a 2θ range of 5–100° 2θ with a scan step of 0.04° on a Bruker D8 Advance powder diffractometer (Bruker-AXS, Karlsruhe, Germany) employing CuKα radiation of U = 40 kV and I = 40 mA and a LynxEye detector (Bruker-AXS, Karlsruhe, Germany). The crystalline phases were defined based on the International Centre for Diffraction Data (ICDD) powder diffraction files.

#### 3.3.6. Temperature-Programmed Reduction with Hydrogen (H_2_-TPR)

H_2_-TPR experiments were performed in the temperature range of 50–900 °C using a Thermo Scientific TPRDO1100 system (Waltham, MA, USA) by a 5% H_2_/Ar gas mixture at a flow rate of 20 cm^3^ min^−1^ and a heating rate of 10 °C min^−1^. Two approaches were applied: normal TPR to 900 °C and isothermal TPR via two modes of after sample reduction at 430 °C for 2 h and after sample reduction at 490 °C for 2 h.

#### 3.3.7. Chemisorption of Hydrogen (H_2_ Chemisorption)

Pulse chemisorption was performed at 40 °C with pure H_2_ and Ar as the carrier gas using a Thermo Scientific TPD/R/O 1100 instrument equipped with a thermal conductivity detector. Prior to chemisorption measurements, all samples underwent identical material preparation steps via in situ treatment. The latter comprised drying in a stream of Ar at 110 °C for 1 h, precursor reduction from 110 to 430 °C at a heating rate of 2 °C/min in a flow of pure H_2_ at 20 cm^3^ min^−1^, and holding for 5 h under hydrogen flow at 430 °C. Then, cooling in Ar to 425 °C was performed to remove hydrogen from the surface and held at the same temperature for 2 h, followed by cooling down to 40 °C also in the Ar stream. Subsequently, pulse chemisorption of each sample was conducted in 5-min intervals using 10 pulses of pure H_2_ at a constant temperature of 40 °C in the argon flow. The calculation of both hydrogen consumption via chemisorption and nickel dispersion was performed using TPD/R/O 1100 software version 3.0.2 (Thermo, Waltham, MA, USA).

### 3.4. Reduction of the Precursors

#### 3.4.1. Ex Situ Reduction

The ex situ reduction procedure was performed to investigate the active form of the catalysts. For this purpose, after the actual reduction, the catalysts were impregnated with refined oil to avoid metallic nickel oxidation by air. The ex situ reduction was done in a quartz reactor at 430 °C for 120 min in hydrogen gas of 99.999% purity at a flow rate of 20 cm^3^ min^−1^ and heating rate of 3.0 °C min^−1^. After finishing the reduction, the reactor was cooled down to a temperature of 40 °C in the H_2_ flow, and the gas in the reactor was switched to argon of 99.999% purity. The reduced samples were denoted as Ni-red, MgNi-red, CeMgNi-red, and MgNi-Ce-red.

#### 3.4.2. Reduction/Activation

Prior to the hydrogenation reaction, preliminary dried precursors at 120 °C for 20 h were activated by dry reduction with hydrogen gas of 99.999% purity in a quartz reactor at a flow rate of 10 L h^−1^ and heating rate of 1.5 °C min^−1^ from 120 °C up to 430 °C and held constant for 5 h. After completion of the reduction followed by cooling to room temperature in the H_2_ flow, the gas in the reactor was switched to argon of 99.999% purity. The reduced precursors were then impregnated with argon-purged paraffin oil to prevent oxidation of the metallic nickel due to its high pyrophoricity. At the end, after the filtration of excess paraffin oil, the obtained catalysts were collected.

### 3.5. Catalyst Performance Evaluation via Partial Hydrogenation of Sunflower Oil

Hydrogenation of commercially available edible sunflower oil was performed in a 1.5 L glass reactor (Parr Instrument, Moline, IL, USA) connected to a computer, an F-201C mass flow controller, and an F-502C pressure meter (Bronkhorst, Ruurlo, The Netherlands). The reactor was connected to a hydrogen source, and the H_2_ pressure was kept constant during the process. For all catalytic tests, the experimental conditions were the same: oil mass-900 g, catalyst concentration-0.06 wt.% Ni relative to the oil amount, stirring rate of 1200 rpm, hydrogenation temperature of 160 °C, and H_2_ pressure of 0.2 MPa. Prior to heating, the reactor containing oil was flushed several times with nitrogen of 99.999% purity to remove the present oxygen and flushed again with hydrogen. The operating temperature of the system was reached in approximately 120 min and maintained for a further 60 min before stirring onset, thus allowing the system to equilibrate.

Hydrogenation activity of the studied catalysts was evaluated by measuring changes in the refractive index (RI) at 50 °C of the starting oil and periodically collected partially hydrogenated oils in accordance with ISO 6320 [60] using a RX-5000α automatic digital refractometer (ATAGO CO, LTD, Tokyo, Japan). Based on our own experience, a temperature of 50 °C allows an unhindered RI measurement of all the collected hydrogenated oils, since the hydrogenation process raises the sample melting temperature.

### 3.6. Fatty Acid Composition

Fatty acid composition of the starting and partially hydrogenated sunflower oils was determined using a Thermo Scientific Trace GC Ultra gas chromatograph equipped with an FID and a TriPlus auto sampler. Triglycerides of the starting and partially hydrogenated oils were firstly converted into their fatty acid methyl esters (FAME) following the standard procedure of the AOCS method [61]. Split injection (split ratio of 1:80) of the prepared FAME samples was performed with helium as the carrier gas at a flow rate of 0.8 mL min^−1^. The column temperature (fused silica capillary column HP-88, 100 m × 0.25 mm i.d. with a 0.20 m film thickness, J&W Scientific—Agilent, Santa Clara, CA, USA) was kept at 170 °C over 75 min of analysis time. Injection port and detector temperatures were 240 and 250 °C, respectively. Individual correction coefficients and concentration calculations were based on previous analysis of the standard mixtures (AOCS#1FAME standard mix—Fame Analysis Restek Restek, Bad Homburg, Germany). Measurements were taken in two repetitions. The results of the fatty acid composition obtained from GC analysis were used for iodine value (IV) calculations. The applied GC method allowed the determination of each individual fatty acid present in the starting oil, as well as in the partially hydrogenated samples. Irrespective of the position of the double bonds, all fatty acids with two double bonds (*cis*, *cis*) in the chain were summarized as C18:2-*cis*. The designation of C18:2-*trans* represents all fatty acids with two double bonds and at least one *trans* conformation, also irrespective of the position of the double bonds in the chain. For monounsaturated acids, the same notation was used, which summarized all *cis* species as C18:1-*cis* and all *trans* species as C18:1-*trans*.

## 4. Conclusions

Based on the explorations presented in this paper, the following conclusions can be drawn:

- Metallic Ni accessibility can be ensured via the joint application of Mg and CeO_2_ as components of the Ni/SiO_2_ catalyst. Design reconstruction of the structure and surface properties positively affects the hydrogenation activity and *cis*/*trans* selectivity of Ni/SiO_2_ catalysts.
- Structure engineering of the vegetable oil hydrogenation Ni/SiO_2_ catalyst via CeO_2_ can be achievable.
- Application of the ceria dopant in Ni/SiO_2_ composition is a promising approach to the improvement of hydrogenation activity, as well as for *cis*/*trans* selectivity, and regulation of the *cis* and *trans* FA content in products of the sunflower oil partial hydrogenation process.

In summary, these investigations extend beyond the scope of this study and will be the subject of our future research.

## Figures and Tables

**Table 1:** Surface chemical composition from the map sum spectrum at magnification 100×.

Spectrum Label (100× Mag)	Component Concertation (wt.%)				Ni/Si *
	Ni	Mg	Ce	O	
Ni-red	51.54	-	-	33.4	3.46
MgNi-red	45.18	1.37	-	33.31	2.26
CeMgNi-red	46.95	1.92	0.9	34.25	3.00
MgNi-Ce-red	46.41	1.85	0.35	34.56	2.82

* Surface Ni/Si ratio of reduced catalysts by SEM-EDS experiments.

**Table 2:** XPS binding energies of the studied reduced catalysts.

Reduced Sample	Ni2p_3/2_ (eV)			Mg1s (eV)	Ce3d_5/2_ (eV)	O1s (eV)	Si2p (eV)
	* Metallic Ni	* Ni^2+^	Ni^2+^/Ni^0^				
Ni-red	852.4(27.7)	856.2(72.3)	2.6			532.5	103.2
MgNi-red	852.9(29.9)	856.4(70.1)	2.3	1304.3		532.5	103.6
CeMgNi-red	852.9(20.7)	861.9(79.3)	3.8	1304.9	not registered	532.9	103.6
MgNi-Ce-red	852.9(27.7)	856.6(72.3)	2.6	1303.8	not registered	532.9	103.8

* Component area as percentage is reported in parentheses.

**Table 3:** XPS surface concentrations.

Reduced Sample	Surface Concentration (at. %)				Ni/Si *
	Ni	Mg	O	Si	
Ni-red	11.0	−	60.9	28.1	0.39
MgNi-red	12.8	2.2	59.5	25.5	0.50
CeMgNi-red	10.0	1.4	62.3	26.3	0.38
MgNi-Ce-red	10.1	2.3	65.9	21.7	0.47

* Relative surface Ni dispersion of the reduced catalysts.

**Table 4:** H_2_ chemisorption data on reduced precursors.

Reduced Sample	SSA_Ni_ (m^2^ g^−1^)	D_Ni_ (%)	Ni Size (nm)	
			Cubic Model	Hemisphere Model
Ni-red	42.7	6.4	13.1	7.9
MgNi-red	63.8	9.6	8.8	5.3
CeMgNi-red	59.0	8.9	9.5	5.7
MgNi-Ce-red	59.3	8.9	9.5	5.7

**Table 5:** Fatty acid composition in the partial hydrogenated sunflower oil at IV = 80. FA content of the starting sunflower oil (mol%): C16:0 = 7.2; C18:0 = 4.0; C18:1-*cis* = 26.0; C18:1-*trans* = 0.02; C18:2-*cis* = 62.2; C18:2-*trans* = 0.06; C18:3-*cis* = 0.10; C20:0 = 0.22; C22:0 = 0.20.

Catalyst	Fatty Acid Composition (wt.%)						
	C18:0	C18:1^a^	C18:2^b^	C18:1-*cis*	C18:2-*cis*	C18:1-*trans*	C18:2-*trans*
Ni-red	22.6	46.9	22.9	32.0	20.4	14.9	2.5
MgNi-red	17.0	58.1	17.5	35.5	14.3	22.6	3.2
CeMgNi-red	11.7	68.4	12.2	44.9	7.3	23.5	4.9
MgNi-Ce-red	13.1	66.0	13.4	43.4	8.4	22.6	5.0

C18:1^a^ = C18:1-*cis* + C18:1-*trans*; C18:2^b^ = C18:2-*cis* + C18:2-*trans*.

**Table 6:** Relative ratio of the change in the C18:1-*cis* content to the change in the content of *trans* isomers for partially hydrogenated oil with an iodine value of 80.

Catalyst	Relative Ratio of the Change C_18:1-*cis*_ Content to C_18:1-*trans*_ Content	Relative Ratio of the Change C_18:1-*cis*_ Content to All *trans* Content
	(C_18:1-*cis*_ − C_18:1-*cis*-*Int*_)/(_18:1-*trans*_ − C_18:1-*trans-Int*_)	(C_18:1-*cis*_ − C_18:1-*cis*-*Int*_)/∑C*_18:x-__trans_ −* C*_18:x-__trans-Int_*)
Ni-red	0.40	0.35
MgNi-red	1.57	1.38
CeMgNi-red	1.91	1.59
MgNi-Ce-red	1.92	1.58

## Data Availability

The original contributions presented in the study are included in the article/Supplementary Materials, and further inquiries can be directed to the corresponding author/s.

## Supplementary Materials

The following supporting information can be downloaded at https://www.mdpi.com/article/10.3390/ijms25147585/s1.
