# Supplementary material for: Structure Engineering of Ni/SiO2 Vegetable Oil Hydrogenation Catalyst via CeO2
Source: Int J Mol Sci. 2024 Jul 10;25(14):7585. doi: 10.3390/ijms25147585 (PMC11276988; doi:10.3390/ijms25147585)
Supplement: Supplementary file 1 [file ijms-25-07585-s001.zip › ijms-3057881-supplementary.pdf]

International Journal of *Molecular Sciences*

Supplementary material

Structure Engineering of Ni/SiO_2 Vegetable Oil Hydrogenation Catalyst Via CeO_2

Margarita Gabrovska [1], Dimitrinka Nikolova [1,*], Vojkan Radonjić [2], Daniela Karashanova [3], Aleksandra Baeva [4], Tsvetomila Parvanova-Mancheva [1], Peter Tzvetkov [4], Evangeliya Petrova [1], Gabriella Zarkova [1] and Jugoslav Krstić [2,*]

1 Institute of Catalysis, Bulgarian Academy of Sciences, 1113 Sofia, Bulgaria; margo@ic.bas.bg (M.G.); tsveti@ic.bas.bg or mila_parvanova@abv.bg (T.P.-M.); evpetrova@ic.bas.bg (E.P.); gzarkova@ic.bas.bg (G.Z.)
2 Institute of Chemistry, Technology and Metallurgy, Department of Catalysis and Chemical Engineering, University of Belgrade, 11006 Belgrade, Serbia; vojkan.radonjic@ihtm.bg.ac.rs
3 Institute of Optical Materials and Technologies, Bulgarian Academy of Sciences, 1113 Sofia, Bulgaria; dkarashanova@yahoo.com
4 Institute of General and Inorganic Chemistry, Bulgarian Academy of Sciences, 1113 Sofia, Bulgaria; baeva@svr.igic.bas.bg (A.B.); tzvetkov@svr.igic.bas.bg (P.T.)
* Correspondence: dimi@ic.bas.bg (D.N.); jugoslav.krstic@ihtm.bg.ac.rs (J.K.); Tel.: +359-2-979-3578 (D.N.); +381-11-2630213 (J.K.)

Table S1. Catalyst composition as-synthesized precursors determined by XRF analysis.

As-synthesized	Component concertation (wt. %)			
precursors	Ni	Si	Mg	Ce
Ni	38.77	23.56	-	-
MgNi	26.28	22.73	0.494	-
CeMgNi	34.50	21.02	1.529	0.4283
MgNi-Ce	36.67	22.20	1.494	0.1452

The actual composition of as-synthesized precursors is determined by X-Ray Fluorescence spectroscopy (XRF) of the analysed area of 13 mm and the data present the average values. The deviation from theoretical values is related to the effect of X-ray depth penetration as well as the structure reconstruction and formation of new Mg-and Ce-included formations, provoking bulk redispersion.

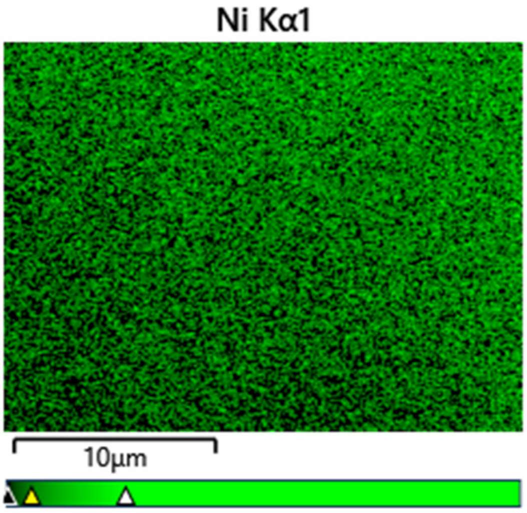

(**a**)

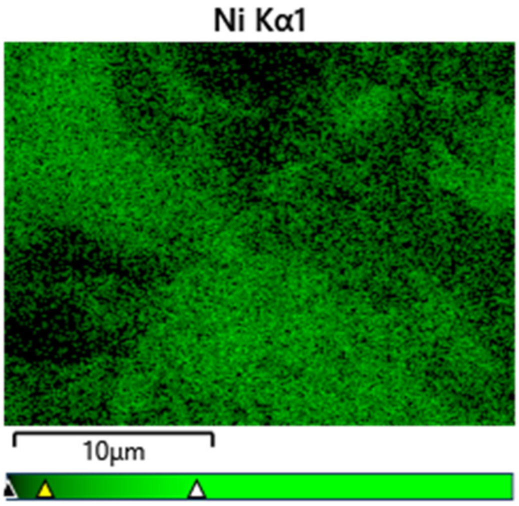

(**b**)

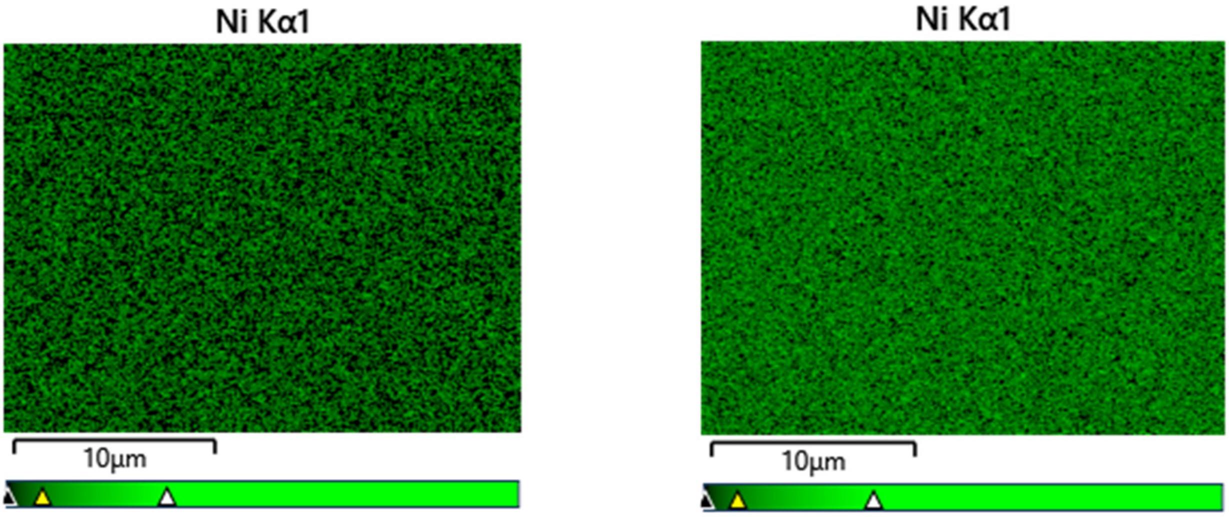

(**c**) (**d**)

Figure S1. EDS elemental mapping of Ni at magnification 2000×: (**a**) Ni; (**b**) MgNi; (**c**) CeMgNi and (**d**) MgNi-Ce.

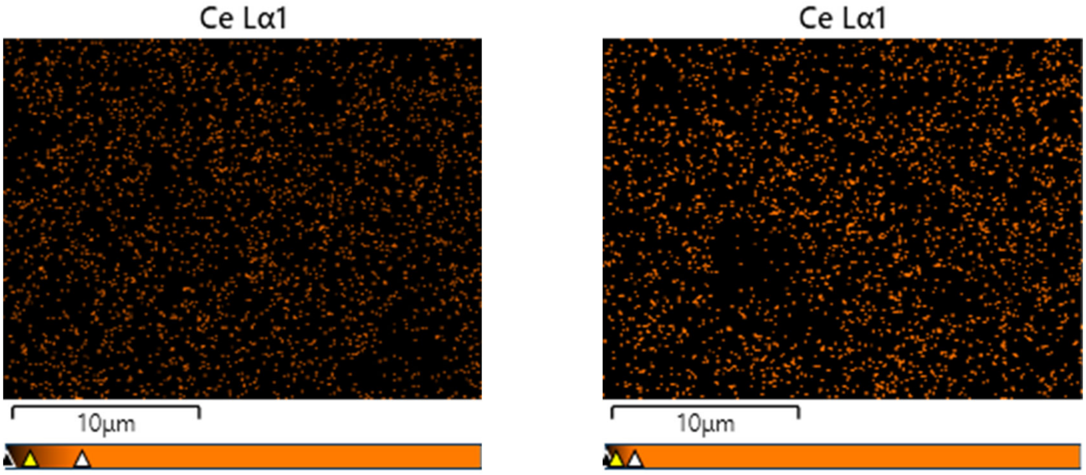

(**a**) (**b**)

Figure S2. EDS elemental mapping of Ce at magnification 100×: (**a**) CeMgNi-red; (**b**) MgNi-Ce-red.

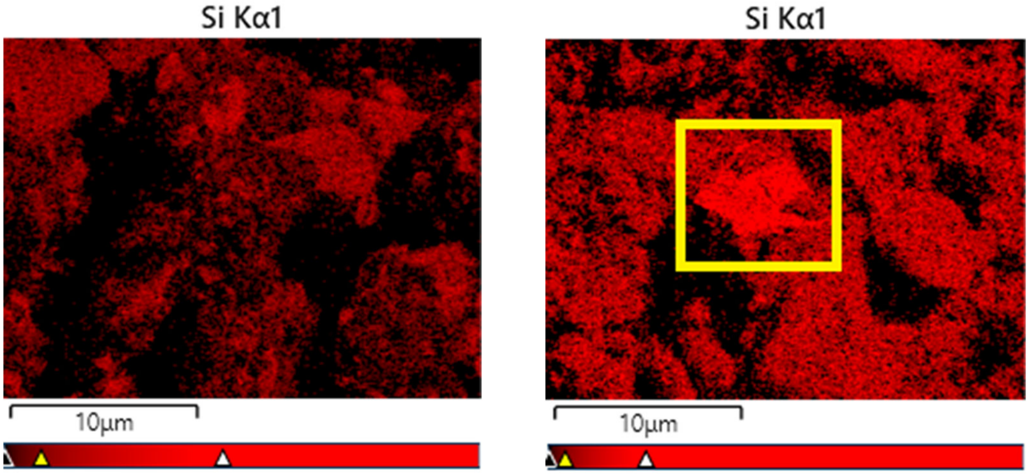

(**a**) (**b**)

Figure S3. EDS elemental mapping of Si at magnification 100×: (**a**) MgNi-red; (**b**) MgNi-Ce-red.

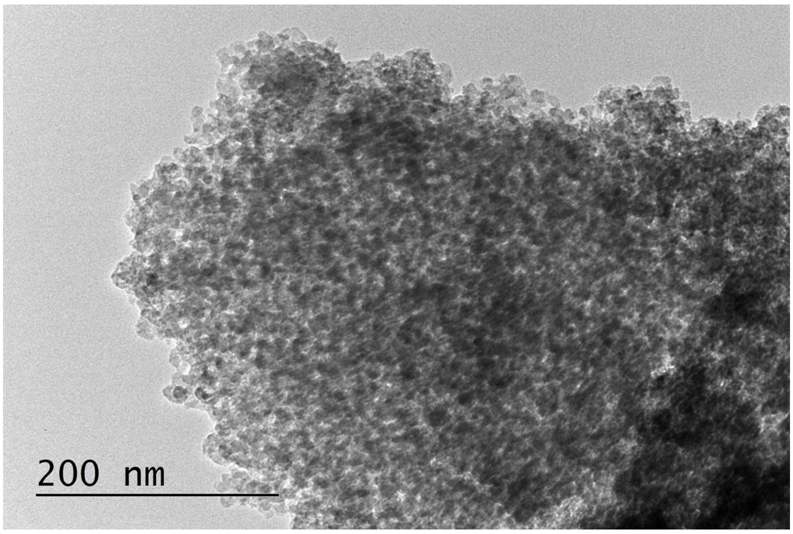

Figure S4. TEM image of as-prepared CeO2-modified silica gel (CeSIG) at magnification 40,000×.

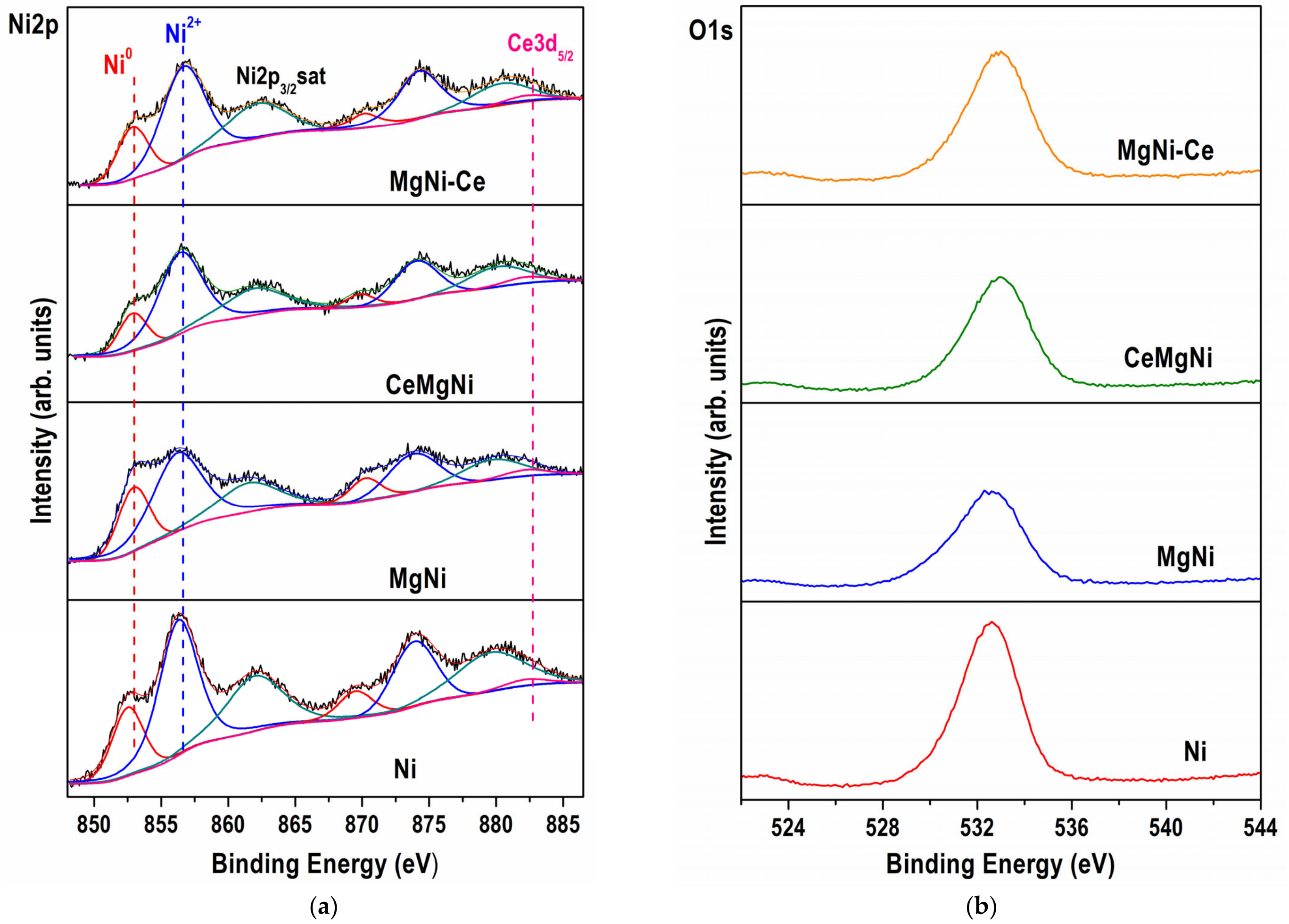

Figure S5. XPS spectra of reduced catalysts: (**a**) Ni2p photoelectron region; (**b**) O1s photoelectron region.

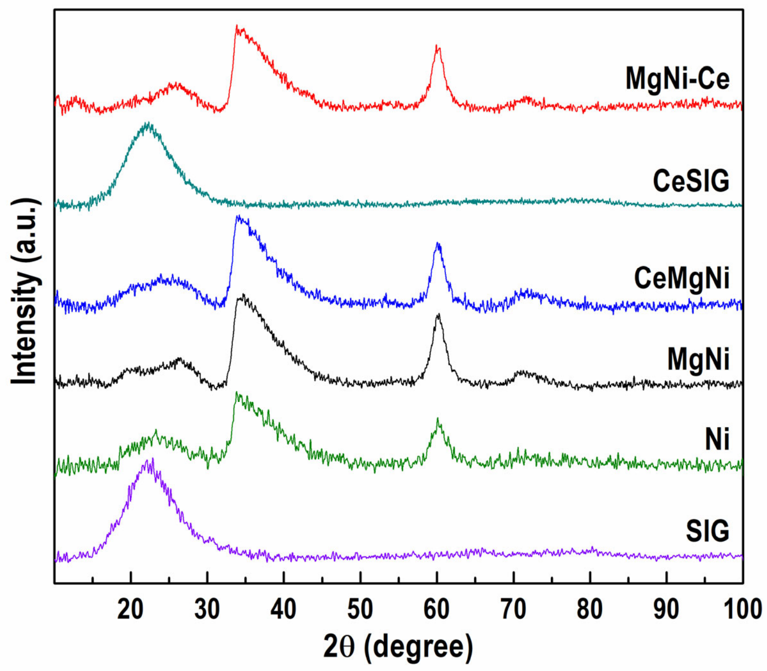

Figure S6. PXRD patterns of the supports and as-prepared catalyst precursors

PXRD patterns of the both supports (Figure S4) displayed reflections characteristic of amorphous silica. The silica halo peak situated at 2θ ≈22° represented the quartz phase in the hexagonal crystal symmetry (Quartz, SiO_2, ICDD-PDF file 00-046-1045). The diffractograms of the both carriers are very similar in the peak angle location and intensity. The absence of a separate ceria phase registration is explained by the low CeO_2 content in the CeO_2-doped support, CeSIG.

The peaks from the diffractograms of Ni, MgNi, CeMgNi and MgNi-Ce samples are weak and broad, meaning poorly crystallized well-dispersed nickel compounds. Furthermore, the lack of sharp peaks suggests the absence of large crystalline domains or clusters, which is favorable for the metal dispersion during the reduction of catalysts.

The patterns of the Ni sample synthesized on SIG support, designated as Ni comprise reflections attributable to a nickel silicate hydroxide phase in the monoclinic crystal symmetry (Pecoraite, $Ni_3Si_2O_5(OH)_4$, ICDD-PDF file 00-049-1859). PXRD patterns of Mg-doped Ni samples supported on SIG, denoted MgNi, CeMgNi as well as MgNi-Ce, deposited on CeSIG show a simultaneous occurrence of the Pecoraite and Nepouite phases (Ni-Mg silicate hydroxide $(Ni,Mg)_3Si_2O_5(OH)_4$ in the orthorhombic crystal symmetry (ICDD-PDF file 00-025-0524).

The presence of nickel silicate, formed by a strong interaction between Ni and Si, was detected in all studied samples at ≈ 34-35° and ≈ 60° peaks. Moreover, the Ni phyllosilicate phase exhibits different degree of crystallization, more pronounced in MgNi sample.

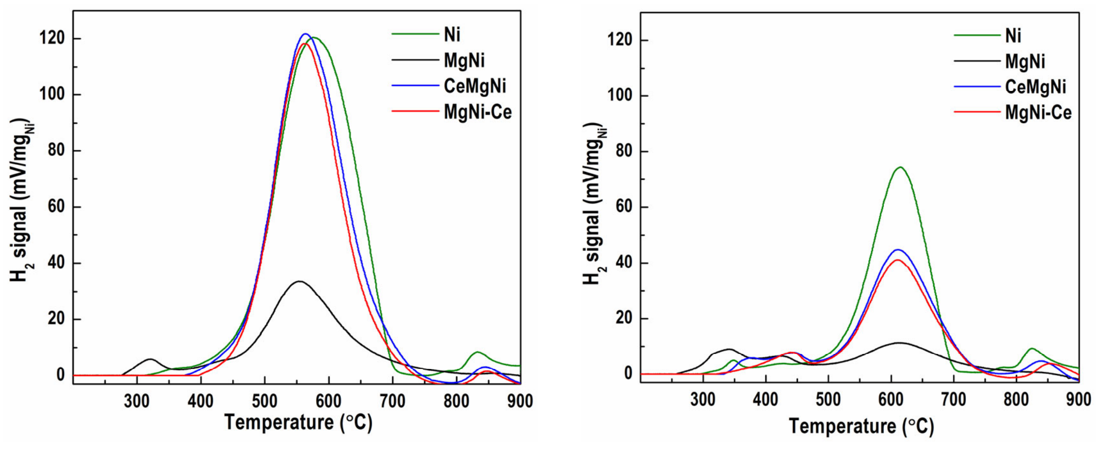

(**a**) (**b**)

Figure S7. Comparative presentation of H_2-TPR profiles of the catalyst precursors: (**a**) after isothermal reduction at 430 °C and (**b**) after isothermal reduction at 490 °C.

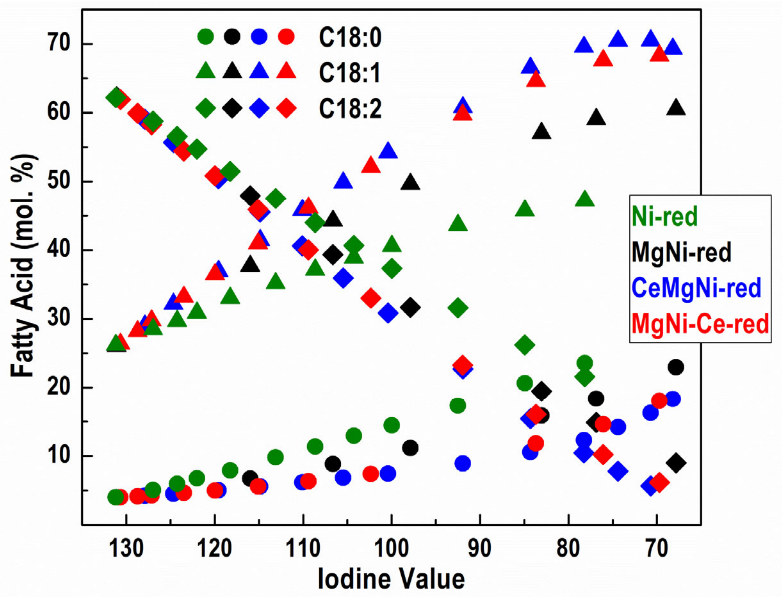

Figure S8. Change in composition of C18:0, C18:1, and C18:2 fatty acids vs. IV during the partial vegetable oil hydrogenation using reduced catalysts.
